# Metformin Restores Tetracyclines Susceptibility against Multidrug Resistant Bacteria

**DOI:** 10.1002/advs.201902227

**Published:** 2020-05-12

**Authors:** Yuan Liu, Yuqian Jia, Kangni Yang, Ruichao Li, Xia Xiao, Kui Zhu, Zhiqiang Wang

**Affiliations:** ^1^ Institute of Comparative Medicine College of Veterinary Medicine Yangzhou University Yangzhou Jiangsu 225009 China; ^2^ Jiangsu Co‐innovation Center for Prevention and Control of Important Animal Infectious Diseases and Zoonoses Yangzhou Jiangsu 225009 China; ^3^ Beijing Advanced Innovation Center for Food Nutrition and Human Health College of Veterinary Medicine China Agricultural University Beijing 100193 China

**Keywords:** antibiotic adjuvant, metformin, multidrug resistant bacteria, tetracycline

## Abstract

Highly persistent incidence of multidrug resistant (MDR) bacterial pathogens constitutes a global burden for public health. An alternative strategy to alleviate such a crisis is to identify promising compounds to restore antibiotics activity against MDR bacteria. It is reported that the antidiabetic drug metformin exhibits the potentiation effect on tetracycline antibiotics, particularly doxycycline and minocycline, against MDR *S. aureus*, *E. faecalis*, *E. coli*, and *S. enteritidis*. Mechanistic studies demonstrate that metformin promotes intracellular accumulation of doxycycline in tetracycline‐resistant *E. coli*. In addition, metformin boosts the immune response and alleviates the inflammatory responses in vitro. Last, metformin fully restores the activity of doxycycline in three animal infection models. Collectively, these results reveal the potential of metformin as a novel tetracyclines adjuvant to circumvent MDR bacterial pathogens and to improve the treatment outcome of recalcitrant infections.

## Graphical abstract

This study demonstrates the huge potential of the antidiabetic drug metformin as tetracyclines adjuvant both in vitro and in vivo, providing a new regimen to treat tetracycline‐resistant bacteria. Evidence show that metformin acts by disrupting bacterial membrane potential and undermining the functions of the efflux pump. These mechanisms work together to promote the intracellular accumulation of tetracyclines, thereby overcoming intrinsic resistance.

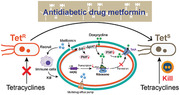

## 1 Introduction

Antibiotics dramatically reduce the deaths caused by severe bacterial infections.^[^ ^1^ ^]^ However, the overuse and misuse of antibiotics inevitably results in the emergence of antibiotic resistance.^[^ ^2^ ^]^ Recently, novel multidrug resistant genes derived from humans, animals and other origins are constantly characterized, including the transferable genes encoding New Delhi metallo‐beta‐lactamase (NDM),^[^ ^3^ ^]^ MCR,^[^ ^4^ ^]^ and Tet(X3/X4)^[^ ^5^, ^6^ ^]^ that mediate resistance to carbapenems, colistin, and tigecycline, respectively. Alarmingly, such crisis is likewise accompanied by a decline in the development of new antibiotics since 1970s, threatening the convenient therapeutic options in the postantibiotic era.^[^ ^7^, ^8^ ^]^

Despite the growing resistance, tetracycline antibiotics remain among the most widely used antibiotics in clinic and agricultural settings.^[^ ^9^ ^]^ Indeed, tetracyclines ranked in one of the top three antibiotics of the clinical prescriptions in the United States in 2010.^[^ ^10^ ^]^ Furthermore, other derivatives including doxycycline, minocycline and tigecycline were continuously introduced into clinical practices. These tetracyclines have the broad‐spectrum activity against both Gram‐positive and Gram‐negative bacteria. Compared to other antibiotics, tetracyclines such as doxycycline and minocycline have better tissue permeability, with high oral bioavailability and low price.^[^ ^11^, ^12^ ^]^ However, tetracycline resistance has been found to be very common in bacteria (above 80% resistance rate).^[^ ^13^ ^]^ The emergence of tetracycline‐resistant pathogens has seriously reduced their efficacies. In addition, a recent study firstly reveals the rapid dynamics of resistance acquisition in susceptible *Escherichia coli* via transferable plasmids encoding the tetracycline‐efflux pump TetA.^[^ ^14^ ^]^ Therefore, innovative cost‐effective strategies warrant to tackle the crisis of tetracycline resistance. The antibiotic adjuvant strategy is a promising approach to extend the lifespan of existing antibiotics through inhibiting bacterial resistance or enhancing antibiotic killing.^[^ ^15^, ^16^ ^]^

To explore the effective antibiotic combinations, we therefore tested the activity of 158 U.S. Food and Drug Administration (FDA)‐approved compounds with doxycycline against MDR *E. coli*. We find that metformin, an oral hypoglycemic agent that widely used as a first‐line therapy for type 2 diabetes,^[^ ^17^ ^]^ remarkably potentiates the activity of doxycycline against a variety of *tet*(A)‐positive resistant pathogens. Further investigations show that metformin disrupts the electrical potential (Δ*ψ*) in *E. coli* and promotes the intracellular accumulation of doxycycline, thus overcoming intrinsic resistance. In addition, metformin modulates host immune responses to infections, including the recruitment of neutrophils and mitigation of inflammatory responses. Collectively, these data demonstrate the potential of metformin as an adjuvant therapy to treat tetracycline‐resistant bacteria associated infections.

## 2 Results

### 2.1 Metformin Potentiates Tetracyclines against MDR Bacteria

**Figure 1:**
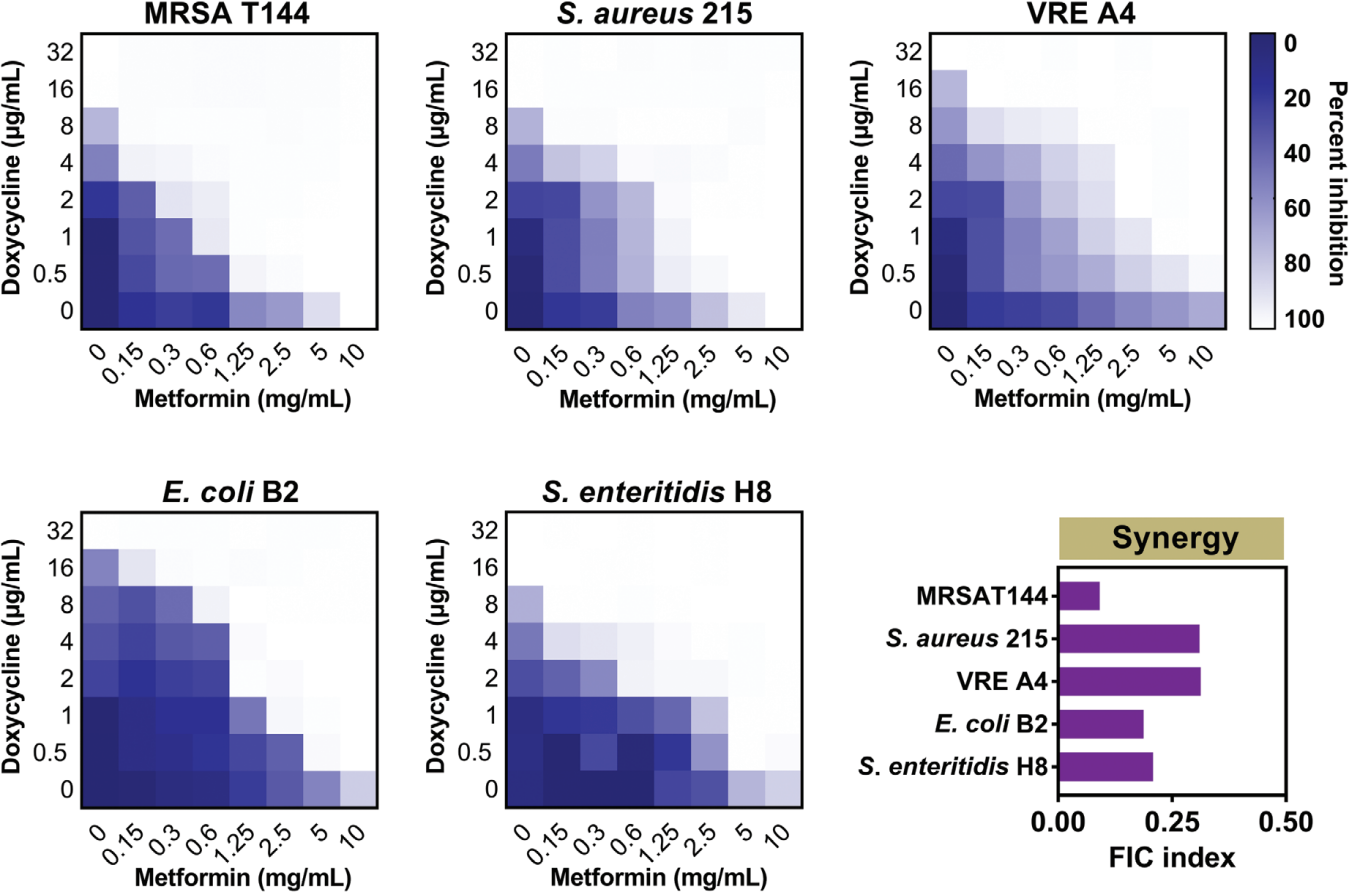
Metformin drastically potentiates doxycycline activity against various multidrug resistant bacteria. Chequerboard broth microdilution assays between metformin and doxycycline against MRSA T144, *S. aureus* 215, VRE A4, *E. coli* B2, and *S. enteritidis* H8. Dark‐blue regions represent higher cell density and lower inhibition rate of combinational treatment. Data represent the mean OD (600 nm) of two biological replicates. *X‐* and *Y*‐axes of figures were presented as log2 scale. The FIC indices were calculated at one quarter of MICs of metformin (2.5 mg mL^−1^ for MRSA T144 and *S. aureus* 215, 5 mg mL^−1^ for VRE A4, *E. coli* B2, and *S. enteritidis* H8). Synergy is defined as an FIC index of ≤ 0.5.

Using the *tet*(A)‐positive *E. coli* B2 as a model strain, which is resistant to almost all tetracyclines except tigecycline, we measured the synergistic activity between doxycycline and 158 FDA‐approved compounds from Prestwick Chemical Library (http://www.prestwickchemical.com) by monitoring bacterial growth curves during 24 h (Figure S1, Supporting Information). The primary screening identified 15 hits (9.49%) that had synergistic activity with doxycycline against *E. coli* B2 (Table S2, Supporting Information). Consistently, some hits including benserazide (a DOPA decarboxylase inhibitor) and loperamide (an opioid receptor agonist) have been previous reported as potential minocycline adjuvants in *P. aeruginosa*.^[^ ^18^ ^]^ Of these hits, metformin was found to exhibit the most potent synergistic effect with an inhibition rate above 90%, whereas metformin alone displayed weak direct antibacterial activity (minimum inhibitory concentration (MIC) ≥ 10 mg mL^−1^). Metformin has been widely used in the clinic for the treatment of type 2 diabetes mellitus.^[^ ^19^ ^]^ Thus, we focused on the potential of metformin as a tetracyclines adjuvant in the subsequent studies. To determine whether this combination is also applicable in other doxycycline‐resistant pathogens, we evaluated the synergistic activity of metformin combined with doxycycline in three Gram‐positive and two Gram‐negative bacteria by chequerboard broth microdilution assays. These pathogens carry *tet*(A) gene and are phenotypically resistant to doxycycline with the MIC values of 16–32 µg mL^−1^ (**Figure** **1** and Table S3, Supporting Information). Consequently, metformin effectively enhanced doxycycline activity against these hard‐to‐treat superbugs with 32–64 folds (Figure 1 and Table S4, Supporting Information), including colistin‐resistant *E. coli* B2, carbapenem‐resistant *S. enteritidis* H8 and vancomycin‐resistant enterococci (VRE) A4. Interestingly, the potentiation of metformin with doxycycline against tetracycline sensitive bacteria such as *S. aureus* ATCC 29213 and *E. coli* ATCC 25922 was lower (FICI = 0.5) than that in MDR bacteria (Figure S2 and Table S4, Supporting Information), suggesting that the inhibition of resistance is due to the presence of metformin. To further test whether this synergy is tetracycline‐specific, we assessed the potentiation of metformin in combination with different classes of antibiotics and other tetracycline antibiotics against *E. coli* B2. Metformin exhibited the synergy with all tetracycline antibiotics except tigecycline, whereas had no synergistic activity with multiple antibiotics including ampicillin (inhibition of cell wall synthesis), colistin (membrane disruption) and ciprofloxacin (targeting DNA synthesis). These results showed that the metformin was a potential adjuvant to tetracyclines, particularly for doxycycline (FICI = 0.078) and minocycline (FICI = 0.188) (Figure S3 and Table S5, Supporting Information).

A critical concern for combinational therapy in clinic is whether there is increased toxicity of antibiotics together with adjuvants. Thus, hemolysis and cytotoxicity of doxycycline in the presence and absence of metformin to mammalian cells were measured. Addition of high‐level metformin (10 mg mL^−1^) had negligible effects on the hemolysis of doxycycline to red blood cells (RBCs) and cytotoxicity to Chinese hamster ovary (CHO) cells (Figure S4A,B, Supporting Information). Collectively, these results demonstrated that metformin was a promising lead to restore tetracyclines activity.

### 2.2 Metformin Enhances Doxycycline Efficacy and Minimize the Emergence of Resistance

**Figure 2:**
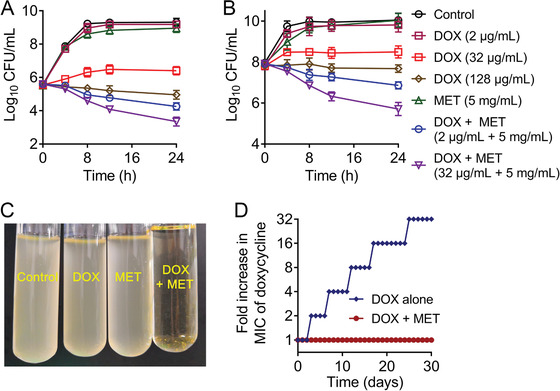
Time‐dependent killing of pathogens by the combination of doxycycline and metformin. *E. coli* B2 were grown to A) early and B) late exponential phases in MHB broth, then treated with PBS, doxycycline (DOX, 2, 32, or 128 µg mL^−1^) or metformin (MET, 5 mg mL^−1^) alone or in combination (DOX + MET, 2 µg mL^−1^ + 5 mg mL^−1^ or 32 µg mL^−1^ + 5 mg mL^−1^). The bacterial CFUs per mL at different time points during 24 h were determined. All experiments were performed three times, and the mean ± SD is shown. C) The combination of doxycycline (128 µg mL^−1^) and metformin (5 mg mL^−1^) leads to bacterial lysis. D) The addition of metformin (2.5 mg mL^−1^, one quarter of MIC) prevents the evolution of doxycycline resistance to *E. coli* ATCC 25922 in vitro. Resistance acquisition during serial passaging in the presence of 0.25 × MIC levels of doxycycline (0.25 µg mL^−1^).

Although chequerboard assays have implied the potentiation of metformin with doxycycline, a direct synergistic bactericidal activity test may strengthen these findings. Thus, we subsequently performed time‐killing experiments on cells at early and late exponential phases after treatments with metformin, doxycycline, and both thereof. We found that either 128 µg mL^−1^ doxycycline (corresponding to fourfold MIC) or 5 mg mL^−1^ metformin showed weak bactericidal activities. By contrast, the combination of doxycycline plus metformin (2 µg mL^−1^ + 5 mg mL^−1^ or 32 µg mL^−1^ + 5 mg mL^−1^) displayed obvious bactericidal activities against bacteria at both early exponential phase (**Figure** **2**A) and late exponential phase (Figure 2B). In comparison, a stronger sterilization effect on early exponential phase bacteria was found, which is consistent with previous observations that quiescent bacteria are relatively hard to eliminate.^[^ ^20^ ^]^ Furthermore, high concentrations of combinational treatments resulted in bacterial lysis (Figure 2C), despite that doxycycline is a bacteriostatic agent.^[^ ^21^ ^]^ In agreement with our observation, a recent report also showed the bactericidal effects of tetracyclines in gut bacteria including *E. coli*.^[^ ^22^ ^]^ To get better understanding of metformin on the development of doxycycline resistance, we performed serial passages of *E. coli* ATCC 25922 with sub‐MIC (0.25 × MIC) of doxycycline in the presence and absence of metformin (2.5 mg mL^−1^, corresponding to a quarter of MIC) during 30 d. Interestingly, we failed to obtain the resistant mutants in the combination group (Figure 2D). In contrast, the doxycycline alone group produces high‐resistant strains with 32‐fold increase of MIC. These results suggested that the combination of doxycycline and metformin could effectively minimize the *de novo* emergence of doxycycline resistance.

### 2.3 Metformin Promotes the Intracellular Accumulation of Doxycycline

**Figure 3:**
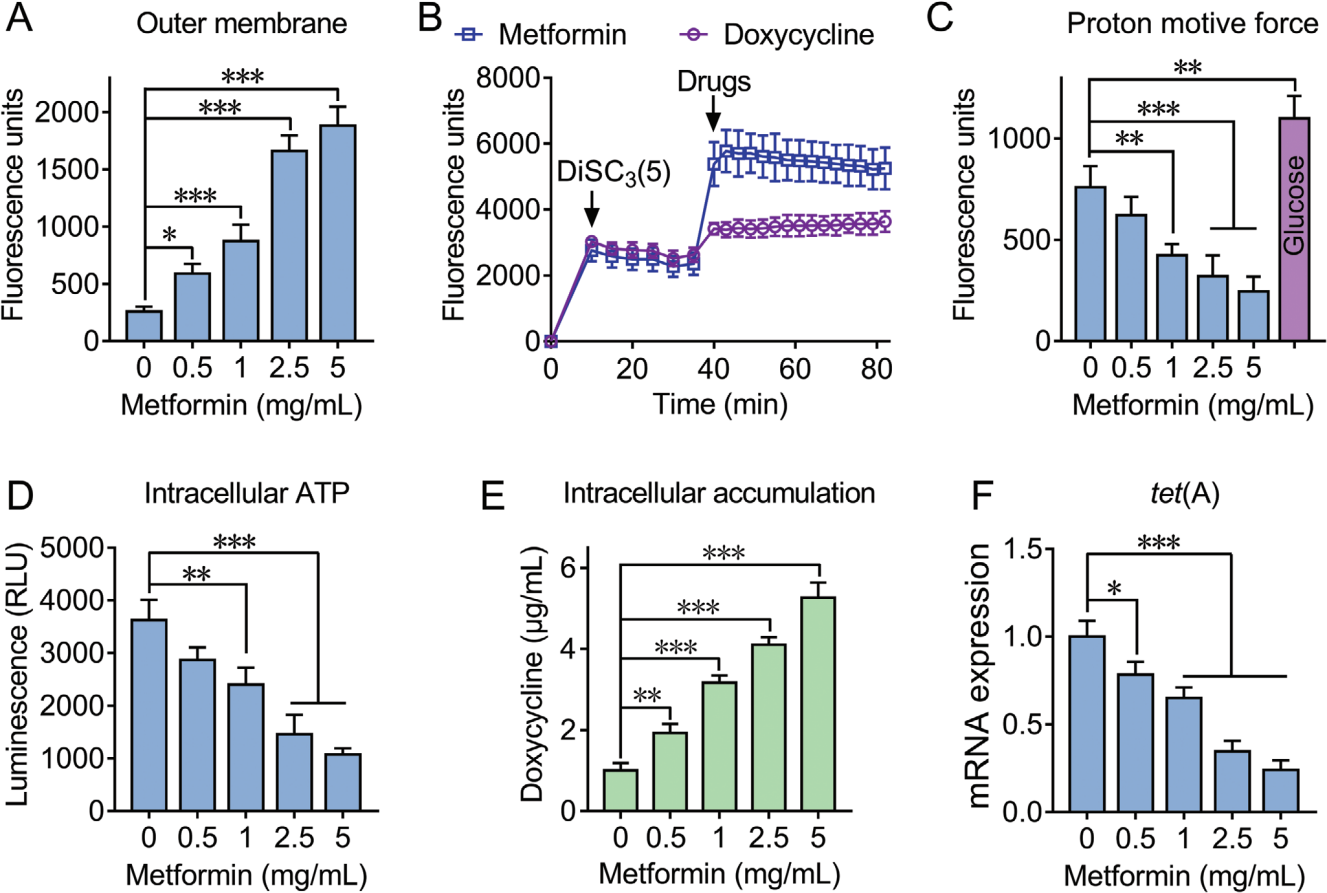
Synergistic mechanisms of doxycycline‐metformin combination. A) Metformin disrupts the outer membrane of *E. coli* B2 by measuring fluorescence intensity of 1‐*N*‐phenylnaphthylamine (NPN) after exposure to increasing concentrations of metformin for 1 h. B) Metformin dissipates membrane potential of *E. coli* B2. DiSC_3_(5) dye was first injected at 10 min followed by self‐quenching and stabilization, then metformin and doxycycline were added at 40 min. The fluorescence units were monitored during 80 min. C) Disruption of proton motive force with increased metformin by monitoring the fluorescence intensity of BCECF‐AM‐probed *E. coli* cells. Glucose was recognized as positive control due to its ability to enhance PMF. D) Decreased production of intracellular ATP in *E. coli* cells treated with metformin, measured by a luciferin‐luciferase bioluminescence assay. E) Increased intracellular accumulation of doxycycline in *E. coli* B2 caused by metformin in a dose‐dependent manner, measured by LC‐MS/MS. Initial concentration of doxycycline was 32 µg mL^−1^. F) Metformin inhibits the transcription of *tet*(A) in a dose‐dependent manner, determined by RT‐PCR analysis. All data are presented as mean ± SD and the significances were determined by nonparametric one‐way ANOVA (*p < 0.05, **p < 0.01, ***p < 0.001).

Having shown that metformin potentiates doxycycline killing against resistant pathogens, we next sought to elucidate the potential mechanisms. Considering that the antibacterial activity of doxycycline is dependent on the inhibition of protein synthesis, thus enough intracellular accumulation of doxycycline is essential for its activity. Consistently, it has been shown that *tet*(A)‐mediated efflux pump is dominant to the resistance of tetracyclines.^[^ ^23^ ^]^ Thus, we hypothesized that metformin might promote the intracellular accumulation of doxycycline. To this end, we first tested the effect of metformin on the permeability of outer membrane and cytoplasmic membrane through fluorescence intensity analysis. 1‐*N*‐phenylnaphthylamine (NPN), a hydrophobic fluorescent probe that releases fluorescence when interact with the hydrophobic parts of phospholipid bilayer,^[^ ^24^ ^]^ was used to monitor the permeability of outer membrane (OM). We found that metformin increased the OM permeability of *E. coli* B2 in a dose‐dependent manner (**Figure** **3**A). In addition, the supplement of Mg^2+^ (10 × 10^−3^ m) abolished the potentiation of metformin to doxycycline, whereas ethylenediamine tetraacetic acid (EDTA) enhanced the synergistic effect (Figure S5, Supporting Information). It has been demonstrated that Mg^2+^ reinforces the bacterial OM, whereas EDTA counteracts such effect.^[^ ^25^ ^]^ Our findings suggested that the disruption of bacterial OM by metformin was crucial for its potentiation to doxycycline in *E. coli*. Consistent with this observation, modest synergistic activities (FICI ranged from 0.375 to 0.5) were observed between metformin and other antibiotics including vancomycin, rifampicin and erythromycin (Table S5, Supporting Information), which could not penetrate the OM of Gram‐negative bacteria.

Subsequently, we used a fluorescent probe propidium iodide (PI)^[^ ^26^ ^]^ to further assess the effect of metformin on bacterial inner membrane (IM). However, no significant increase of fluorescence was observed when probed *E. coli* cells exposed to metformin (Figure S6A, Supporting Information). These results demonstrated that metformin disrupted the integrity of bacterial OM, regardless of bacterial IM. We speculated that metformin might cause dysfunctions in cytoplasmic membrane. To test this, DiSC_3_(5) was used to evaluate the bacterial membrane potential.^[^ ^27^ ^]^ Addition of metformin to DiSC_3_(5)‐probed cells resulted in threefold enhanced fluorescence, whereas doxycycline alone had weak effects on the loss of membrane potential (Figure 3B), suggesting that metformin disrupted the electric potential (Δ*ψ*) of *E. coli*. Previous studies demonstrated that the membrane depolarization is related to the production of reactive oxygen species (ROS) and proton motive force (PMF).^[^ ^28^, ^29^ ^]^ To dissect the roles of ROS and PMF, we subsequently used dyes DCFH‐DA^[^ ^30^ ^]^ and BCECF‐AM,^[^ ^31^ ^]^ respectively. There was no effect on ROS accumulation in *E. coli* treated with metformin (Figure S6B, Supporting Information). Compared to the increased PMF in bacteria supplied with glucose, metformin led to decreased fluorescence (Figure 3C), implying the disruption of PMF. Because PMF is the driving force for ATP synthesis,^[^ ^32^ ^]^ the intracellular levels of ATP also significantly decreased in *E. coli* treated with metformin (Figure 3D). Considering that PMF is critical for the functions of efflux pump,^[^ ^33^ ^]^ we next evaluated the activity of efflux pump in bacteria after exposure to metformin using ethidium bromide (EtBr) as a fluorescent probe. Consistently, decreased efflux of EtBr in *E. coli* B2 and MRSA T144 after incubation with metformin (Figure S7, Supporting Information) was observed. As a result, it showed metformin‐dependent accumulation of doxycycline in *E. coli* through LC‐MS/MS analysis (Figure 3E). Similarly, metformin dissipated membrane potential of *S. aureus* ATCC 29213 in a dose‐dependent manner (Figure S8A,B, Supporting Information). Given that the uptake of aminoglycoside antibiotics is highly dependent on bacterial membrane potential,^[^ ^34^, ^35^ ^]^ thus we next determined the concentrations of kanamycin in *S. aureus* incubated with metformin. Along with the collapse of Δ*ψ* by metformin, a decreased kanamycin uptake in *S. aureus* was observed (Figure S8C, Supporting Information). Consistently, combination of metformin and kanamycin leads to antagonistic interactions both in sensitive bacteria (*S. aureus* ATCC 29213 and *E. coli* ATCC 25922) and resistant bacteria (MRSA T144 and *E. coli* B2) (Figure S9, Supporting Information).

**Figure 4:**
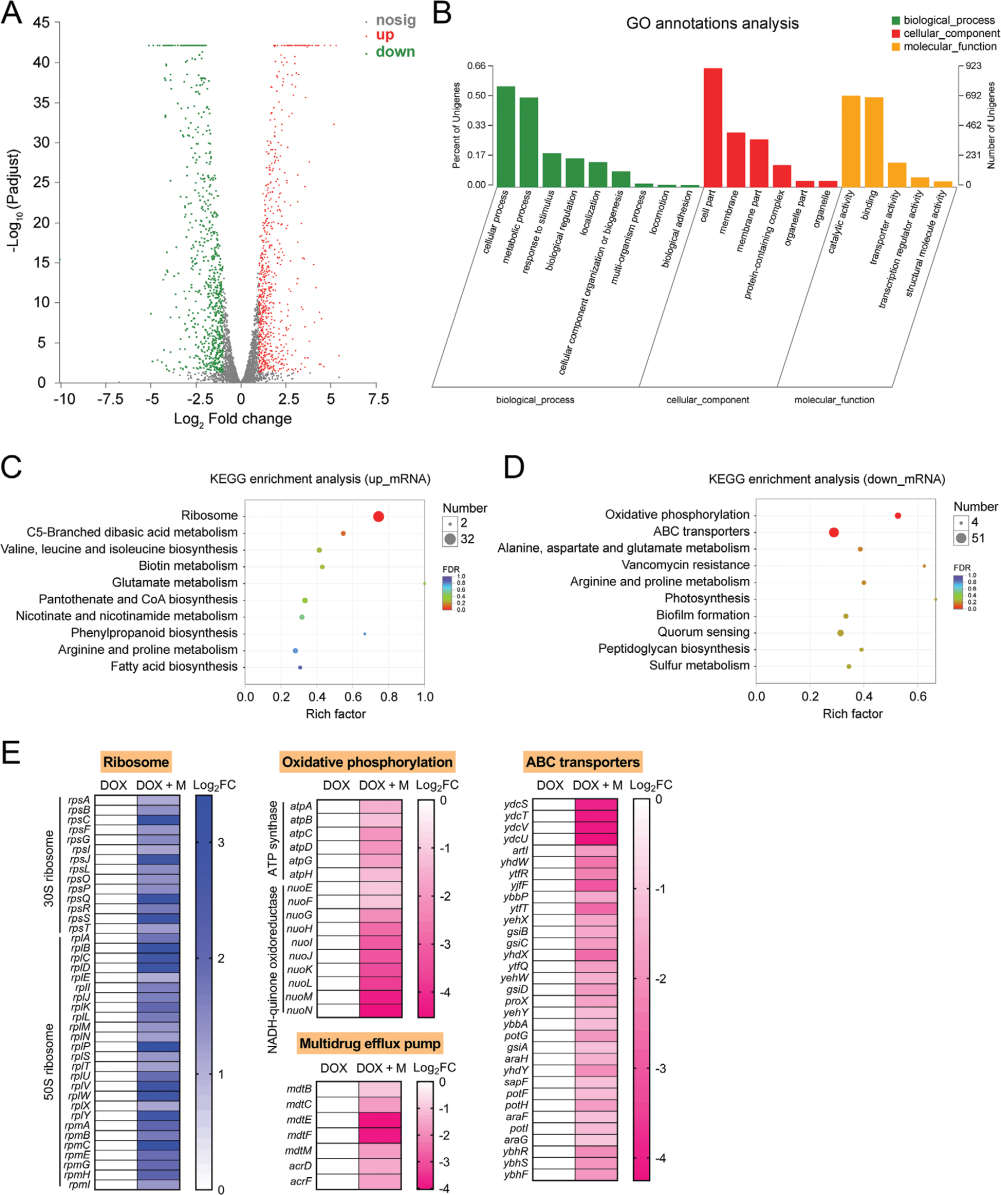
Transcriptome analysis of *E. coli* B2 after exposure to doxycycline alone or the combination of doxycycline plus metformin. A) Volcano plot and B) GO (gene ontology) annotation analysis of the differential expression genes (DEGs) in *E. coli* B2 after exposing doxycycline (16 µg mL^−1^) or the combination of doxycycline (16 µg mL^−1^) plus metformin (5 mg mL^−1^) for 4 h. The *x‐* and *y*‐axes in (A) represent the expression changes and corresponding statistically significant degree, respectively. An adjusted *p*‐value < 0.05 (Student's *t*‐test with Benjamini–Hochberg false discovery rate adjustment) and |log_2_ Fold change| ≥1 were applied as the cutoff for significant DEGs. KEGG (Kyoto Encyclopedia of Genes and Genomes) enrichment analysis of C) upregulated DEGs and D) downregulated DEGs. The 10 most significant enriched pathways are shown. E) Selected differential expression genes involved in ribosome, oxidative phosphorylation, ABC transporters and multidrug efflux pump. Data were presented as means of three biological replicates. DOX, doxycycline alone; DOX + M, the combination of doxycycline and metformin.

Interestingly, we also observed a reduced transcription of *tet*(A) in *E. coli* B2 treated with metformin in RT‐PCR analysis (Figure 3F). To gain a deeper understanding of the molecular mechanisms of metformin and induced gene expression changes at mRNA level, we performed transcription analysis of *E. coli* B2 after exposure to doxycycline or doxycycline‐metformin combination for 4 h. The comparison of treatment with combination to doxycycline alone revealed an up‐regulation of 663 and down‐regulation of 732 differentially expressed genes (DEGs) (**Figure** **4**A). GO annotation analysis showed that these DEGs are correlated with biological processes (e.g., cellular and metabolic process), cellular components (e.g., cell part) and molecular functions (e.g., catalytic activity and binding) (Figure 4B). KEGG enrichment analysis demonstrated that these up‐regulated DEGs were significantly enriched in ribosome synthesis, and down‐regulated DEGs were involved in oxidative phosphorylation and ABC transporters (Figure 4C,D). Specifically, 30S and 50S subunit synthetic genes in bacteria up‐regulated under combinational treatment (Figure 4E). It is plausible that metformin resulted in intracellular accumulation of doxycycline in *E. coli* and thereby inhibited protein synthesis, which is compensated by an up‐regulation of ribosome synthesis related genes. In contrast, oxidative phosphorylation related genes including ATP synthase and NADH‐quinone oxidoreductase significantly down‐regulated (Figure 4E), which was consistent with the decreased ATP level by metformin (Figure 3D). Notably, ABC transporters and multidrug efflux pump associated genes drastically decreased (Figure 4E), implying a weakened functions of efflux pump in *E. coli* by metformin. Collectively, these data demonstrated that metformin promoted intracellular accumulation of doxycycline through disrupting membrane potential as well as outer membrane permeabilization (only in Gram‐negative species), and inhibiting the functions of efflux pump.

### 2.4 Metformin Alleviates the Inflammatory Response Caused by LPS

**Figure 5:**
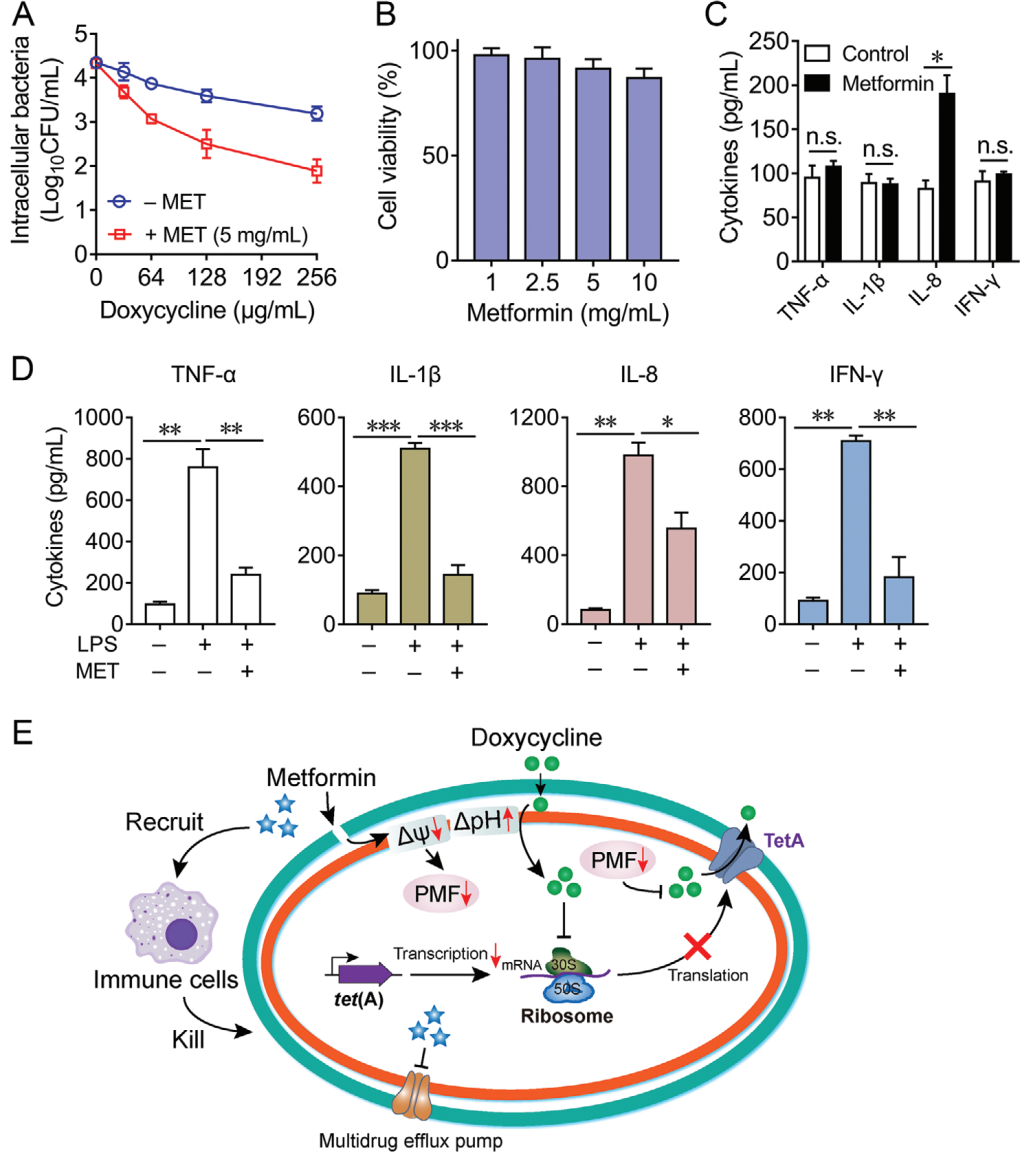
Immunomodulatory functions of metformin in the eradication of resistant pathogens. A) Supplement of metformin (5 mg mL^−1^) in combination with doxycycline (0–256 µg mL^−1^) for 6 h decreases the intracellular bacteria load of *E. coli* B2 in Vero cells, compared with the doxycycline alone group. B) Cytotoxicity of metformin in RAW264.7 cells by WST‐1 assay. RAW264.7 cells were stimulated with metformin (1–10 mg mL^−1^). Absorbance at 450 nm were determined in culture supernatant after 24 h incubation. The *y*‐axis shows the percentage of cell viability to unstimulated cells. C) Effect of metformin on the release of various cytokines in RAW264.7 cells. The cytokines production including TNF‐*α*, IL‐1*β*, IL‐8, and IFN‐*γ* in supernatants after 24 h stimulation was determined by ELISA. Data are presented as mean ± SD from three independent experiments and the significances were determined by unpaired *t*‐test (n.s., not significant, *p < 0.05). D) Metformin alleviates inflammatory response induced by bacterial LPS (1 µg mL^−1^). RAW264.7 cells were pretreated with metformin (5 mg mL^−1^) for 30 min, then stimulated by LPS for 24 h. After incubation, the cytokines in culture samples were monitored by ELISA analysis. All data are presented as mean ± SD from three independent experiments and the significances were determined by nonparametric one‐way ANOVA (*p < 0.05, **p < 0.01, ***p < 0.001). E) Scheme of synergistic mechanisms of metformin in combination with doxycycline against tetracycline‐resistant pathogens. After destroying the outer membrane (only in Gram‐negative bacteria), metformin dissipates membrane potential of cytoplasmic membrane and decreases the proton motive force (in both Gram‐positive bacteria and Gram‐negative bacteria), which subsequently undermines the functions of PMF‐driven efflux pump. To counter this effect, bacteria increase the pH gradient and in turn aid the uptake of doxycycline. These combined actions promote the intracellular accumulation of doxycycline in resistant bacteria. Meanwhile, accumulated doxycycline inhibits the synthesis of bacterial proteins including TetA. In addition, metformin could moderately modulate the immune response by recruitment of neutrophils and control of inflammatory response. Multiple synergistic mechanisms make metformin able to restore tetracyclines activity against resistant pathogens.

It has been suggested that nonobligate intracellular bacteria such as *E. coli* and *S. aureus* could invade and survive in host cells to escape the clearance by host immune defense or antibiotic killing.^[^ ^36^, ^37^, ^38^ ^]^ Thus, we sought to evaluate the efficacy of the combination of metformin and doxycycline in cell infection model. The intracellular bacterial loads in Vero cells exposure to doxycycline in the presence and absence of metformin were analyzed by detecting colony forming units (CFUs) of *E. coli*. The supplement of metformin (5 mg mL^−1^) with doxycycline (256 µg mL^−1^) sharply reduced 99% bacteria (**Figure** **5**A), indicating that this combination was efficacious in eliminating persistent intracellular pathogens.

In addition, we next explored whether metformin possesses immunomodulatory properties like some cationic host‐defense peptides.^[^ ^39^, ^40^ ^]^ To test this, we first evaluated the cytotoxic effect of metformin in RAW264.7 macrophages. We found that metformin showed less than 10% cytotoxicity at 5 mg mL^−1^ (Figure 5B). Thus, a dose of 5 mg mL^−1^ was applied to assess the immunomodulatory activity of metformin. RAW264.7 cells were stimulated with metformin alone (5 mg mL^−1^) for 24 h, and the production of pro‐inflammatory cytokines TNF‐*α*, IL‐1*β*, and IFN‐*γ* as well as the chemokines IL‐8 were determined. Interestingly, metformin selectively led to the nearly twofold increase of IL‐8, which is critical during bacterial infections.^[^ ^41^ ^]^ While, there was no effect on the production of TNF‐*α*, IL‐1*β* and IFN‐*γ* (Figure 5C), implying that metformin might recruit the neutrophils to the sites of infections. Consistently, it has been demonstrated that metformin augments the host immune defense and effectively controls *Mycobacterium tuberculosis* infection.^[^ ^42^ ^]^ After that, we next sought to investigate the effects of metformin on inflammatory responses. Lipopolysaccharide (LPS) was applied to mimic bacterial pathogens‐induced inflammation. Exogenous addition of metformin prior to LPS stimulation in macrophages significantly suppressed the production of four cytokines (Figure 5D). These results together showed that metformin not only specifically potentiated doxycycline activity via the increased accumulation of intracellular antibiotics, but also modulated the immune response against infections (Figure 5E).

### 2.5 Metformin Reverses Doxycycline Resistance In Vivo

**Figure 6:**
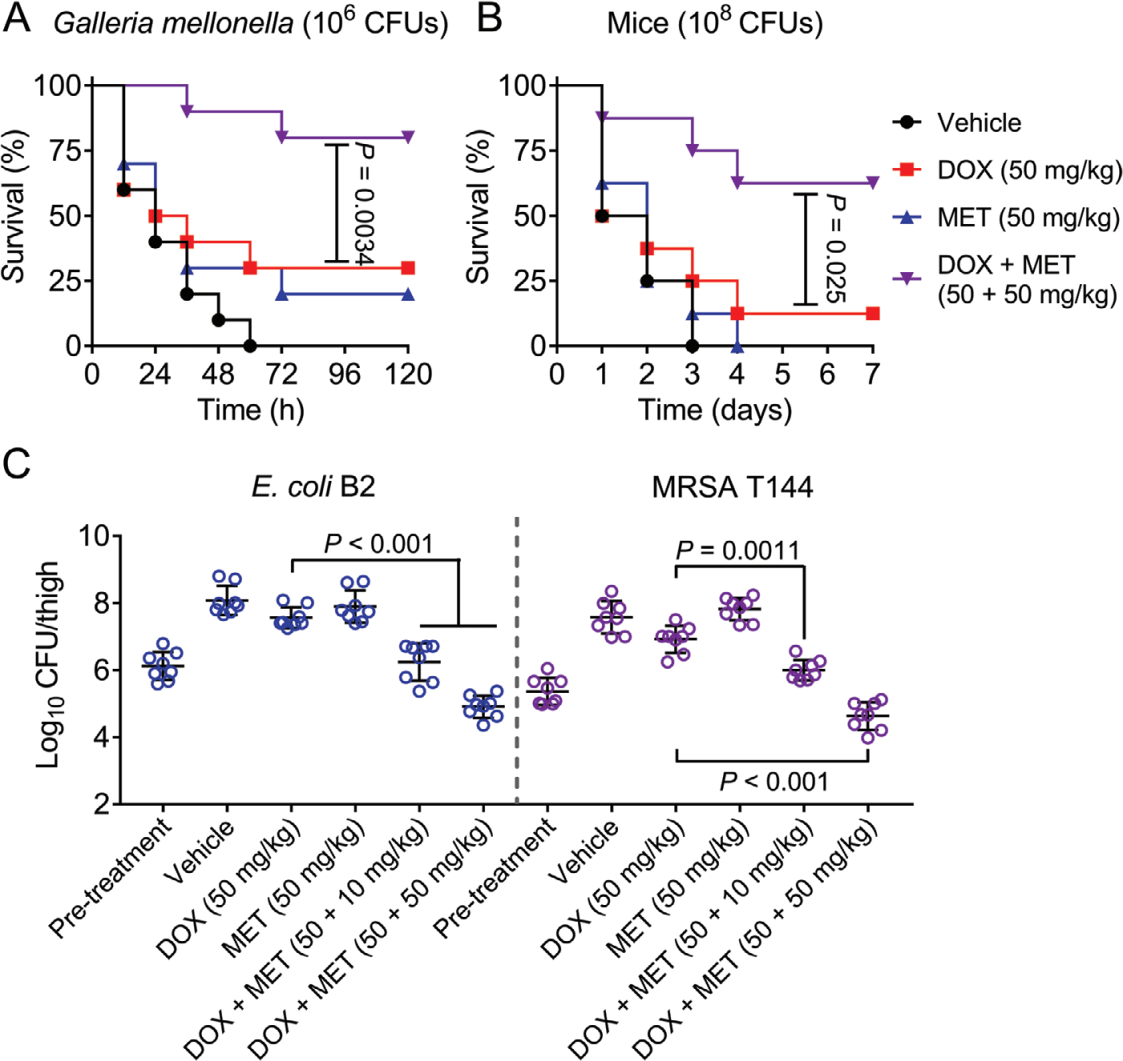
Metformin rescues doxycycline activity in vivo. A) Survival rates of the *G. mellonella* larvae (*n* = 10 per group) infected by *E. coli* B2 (1.0 × 10^6^ CFUs) at the right posterior gastropoda with the treatments of doxycycline (50 mg kg^−1^) or metformin (50 mg kg^−1^) alone or in combination (50 + 50 mg kg^−1^) at left posterior gastropoda (*n* = 10 per group). *p*‐values were determined by log‐rank (Mantel‐Cox) test. B) Survival rates of the female BALB/C mice (*n* = 8 per group) infected by a lethal dose of *E. coli* B2 (1.0 × 10^8^ CFUs) and treated with a single dose of doxycycline (50 mg kg^−1^) or metformin (50 mg kg^−1^) alone or a combination of doxycycline plus metformin (50 + 50 mg kg^−1^), or PBS as vehicle by intraperitoneal injection. *p*‐values were determined by log‐rank (Mantel‐Cox) test. C) Bacterial load of infected thigh muscle in neutropenic mice (*n* = 8 per group) by a nonlethal dose of *E. coli* B2 or MRSA T144 decreased significantly after a single intraperitoneal combination therapy. *p*‐values were determined by Mann–Whitney U test.

After elucidating the potentiation of metformin with doxycycline in bacterial assays and in cell infection model, and its immunomodulatory functions in macrophage cells, we next assessed whether these effects could result in positive outcomes in animal infection models. To confirm this, we tested the in vivo efficacy of the combination of doxycycline and metformin in three preclinical infection models infected with resistant *E. coli* B2 or MRSA T144. First, *G. mellonella* larvae infections models were constructed and used for this evaluation. As shown in **Figure** **6**A, the larvae in vehicle group all died during 72 h and that in doxycycline or metformin alone groups exhibited below 30% survival rate. Compared with the doxycycline administration group, the survival rate of larvae in combination group significantly increased (*p* = 0.0034) and achieved 80% survival after 120 h postinfection. Similarly, expected in vivo efficacy of combination therapy was also observed in a mouse peritonitis infection model. Doxycycline plus metformin treatment obtained survival benefit than the doxycycline group (*p* = 0.025) (Figure 6B). Last, we tested the combinational efficacy in a neutropenic mouse thigh infection model infected with Gram‐negative bacteria (*E. coli* B2) or Gram‐positive bacteria (MRSA T144). Encouragingly, the combinational therapy of doxycycline and metformin (50 + 10 mg kg^−1^) displayed ≈2‐log_10_ reductions in CFUs compared with doxycycline monotherapy. Conceivably, the higher concentration combination (50 + 50 mg kg^−1^) exhibited more potent CFUs reduction of two pathogens (above 3‐log_10_) (Figure 6C). These in vivo efficacious results demonstrated the adjuvant potential of metformin with doxycycline to tackle bacterial infectious diseases caused by growing resistant pathogens.

## 3 Discussion

The emergence and rapid spared of antibiotic resistance in pathogenic bacteria pose a severe threat for public health worldwide.^[^ ^43^ ^]^ Identification of novel adjuvants that restore existing antibiotic efficacy and improve clinical outcomes for patients with infectious diseases has been recognized as a cost‐effective strategy for combating these super bugs.^[^ ^44^ ^]^ In particular, the inhibitors of *β*‐lactamases such as clavulanic acid has been widely used in clinic for decades.^[^ ^45^ ^]^ Recently, statins are found to disassemble bacterial membrane microdomains and disable PBP2a oligomerization, thus restore MRSA susceptibility to penicillin treatment.^[^ ^46^ ^]^ In addition, antiprotozoal drug pentamidine potentiates hydrophobic antibiotic activity against Gram‐negative pathogens through disrupting bacterial outer membrane.^[^ ^47^ ^]^ These existing examples inspire us to investigate the potential of non‐antibacterial agents as potential adjuvants of tetracyclines. Consequently, an FDA‐approved compound metformin was firstly identified as a tetracycline potentiator in our study. Consistent with our finding, a recent clinical study reports the adjuvant potential of metformin with tetracyclines for the treatment of acne vulgaris, but there is complete lack of understanding of the underlying mechanisms.^[^ ^48^ ^]^

Metformin is the only approved hypoglycemic drug for the treatment of type 2 diabetes mellitus in children in partly due to its great safety.^[^ ^49^ ^]^ In recent years, it has also been found to treat cardiovascular disease,^[^ ^50^ ^]^ reduce tumor incidence and mortality,^[^ ^51^, ^52^ ^]^ delay aging,^[^ ^53^ ^]^ reduce heart disease caused by air pollution^[^ ^54^ ^]^ and reverse pulmonary fibrosis.^[^ ^55^ ^]^ However, its potential application in the treatment of bacterial diseases is rarely reported thus far. In this study, we found that metformin effectively potentiates tetracyclines against multiple *tet*(A)‐positive pathogens with an exception of tigecycline. The fact that *tet*(A) only confers low‐level of tigecycline resistance^[^ ^56^ ^]^ may account for such indifference. It is interesting to investigate the underlying molecular mechanisms, which may aid to identify novel tigecycline adjuvants.

In Gram‐negative bacteria, metformin could first destroy bacterial outer membrane, which is highly impermeable and constitutes a barrier for many effective antibacterial agents.^[^ ^57^ ^]^ In light of the observation that Mg^2+^ abolished the potentiation of metformin, we suspected that metformin might disrupt the integrity of outer membrane by displacing the divalent cations such as Mg^2+^, which stabilize bacterial outer membrane.^[^ ^58^ ^]^ In both Gram‐positive bacteria and Gram‐negative bacteria, metformin dissipated membrane potential of cytoplasmic membrane. Similar to loperamide,^[^ ^18^ ^]^ a potential minocycline adjuvant, metformin‐treated bacteria might compensatorily increase the proton gradient, which in turn enhanced the uptake of tetracycline antibiotics. Besides, metformin dramatically undermines the functions of PMF‐driven efflux pump in resistant bacteria. These mechanisms work together to promote the accumulation of tetracycline antibiotics in bacterial cells, thereby overcoming *tet*(A)‐mediated tetracycline resistance.

In addition to direct potentiation activity to tetracyclines, we found that metformin could moderately activate innate immune response. Moreover, metformin alleviated inflammatory responses induced by endotoxins such as LPS. In fact, the immunomodulatory properties of metformin in macrophages have been partially reported. For example, metformin has been found to activate the adenosine monophosphate‐activated protein kinase (AMPK)‐mediated autophagy, which facilitates the formation of autophagosome and provides an effective control of intracellular pathogens.^[^ ^59^ ^]^ Meanwhile, metformin provides a protection against *Legionella pneumonia* through promoting AMPK signaling and mitochondrial ROS production.^[^ ^60^ ^]^ However, more works are still needed to elucidate the underlying mechanisms of immunomodulatory properties of metformin against bacterial infections.

In summary, versatile metformin coupled with satisfied safety, inhibition of resistance development and excellent preclinical data, suggests that metformin represents a promising tetracyclines adjuvant to tackle the clinically relevant pathogenic bacteria. Meanwhile, the identification of metformin encourages us to discover more candidates with collaborative mechanisms as potential antibiotic adjuvants. Nevertheless, more prospective clinical trials to verify the potentiation activity of metformin with tetracyclines in vivo are still required.

## 4 Experimental Section

##### Bacteria and Reagents

All strains used in this study were listed in Table S1 (Supporting Information). Unless otherwise noted, strains were grown in Mueller‐Hinton broth (MHB, Qingdao Hope Bio‐technology) or on MH agar (MHA) plates at 37 °C. CHO, Vero and RAW264.7 cells were grown in Dulbecco's Modified Eagle's Medium (DMEM, Gibco) supplemented with 10% heat inactivated fetal bovine serum (FBS, Invitrogen), 1% (w/v) penicillin‐streptomycin and 1% (w/v) sodium pyruvate (Sigma‐Aldrich, Oakville, Ontario). All antibiotics were obtained from China Institute of Veterinary Drug Control.

##### MIC Assay

MICs of all compounds were determined by the standard broth microdilution method, according to the CLSI 2015 guideline.^[^ ^61^ ^]^ Briefly, drugs were twofold diluted in MHB and mixed with an equal volume of bacterial suspensions containing approximately 1.5 × 10^6^ colony‐forming units (CFUs) mL^−1^ in a clear UV‐sterilized 96‐well microliter plate (Corning, New York, USA). After 18 h incubation at 37 °C, the MIC values were defined as the lowest concentrations of antibiotics with no visible growth of bacteria.

##### Doxycycline Adjuvant Screening

158 FDA‐approved compounds from Prestwick Chemical Library (Prestwick Chemical Inc, Illkirch, France) were screened against *E. coli* B2 (*tet*(A)) in combination with 8 µg mL^−1^ doxycycline (one quarter of MIC). Briefly, doxycycline and/or compounds were diluted in MHB and mixed with an equal volume of bacterial suspensions (1.5 × 10^6^ CFUs mL^−1^) in the 96‐well microliter plate. Then, the real‐time growth curves of *E. coli* B2 in the absence or presence of drugs were monitored during 24 h. MHB medium containing doxycycline (8 µg mL^−1^) with or without bacteria was served as positive and negative controls, respectively. Experiments were performed with three biological replicates. Absorbance at 600 nm of bacterial culture at the time point of 24 h was collected. The inhibition rate (%) was calculated as[(OD_positive control_ − OD_negative control_) − (OD_sample_ − OD_negative control_)] / (OD_positive control_ − OD_negative control_) × 100%. Synergy effect was defined as the inhibition rate of ≥ 50%.

##### Checkerboard Studies

Synergistic activity between compounds and antibiotics, and the fractional inhibitory concentrations (FIC) indices were measured by checkerboard assays.^[^ ^62^ ^]^ Briefly, 100 µL of MHB was dispensed into each well of a 96‐well plate. Antibacterial drugs were diluted along the abscissa while compounds were diluted along the ordinate. Overnight tested bacterial culture was standardized to match a 0.5 McFarland turbidity standard and followed by diluted 1:100 in MHB broth. After incubated at 37 °C for 18 h, the optical density of each wells at 600 nm were determined by an Infinite M200 Microplate reader (Tecan, Männedorf, Switzerland). The FIC index (FICI) was calculated according to the formula as follows^[^ ^63^ ^]^

(1) $$\mathrm{FIC}\;\mathrm{index}=\mathrm{MI}C_{\mathrm{ab}}/\mathrm{MI}C_{a}+\mathrm{MI}C_{\mathrm{ba}}/\mathrm{MI}C_{b}=\mathrm{FI}C_{a}+\mathrm{FI}C_{b}$$

where MIC_a_ is the MIC of compound A alone, MIC_ab_ is the MIC of compound A in combination with compound B, MIC_b_ is the MIC of compound B alone, MIC_ba_ is the MIC of compound B in combination with compound A, FIC_a_ is the FIC index of compound A, and FIC_b_ is the FIC index of compound B. Synergy is defined as an FIC index of ≤ 0.5.

##### Safety Assessment

Effect of metformin on the hemolytic activity of doxycycline was evaluated based on previous report.^[^ ^64^, ^65^ ^]^ Briefly, 8% sheep blood cells that prepared from fresh sterile defibrinated sheep blood, was equal‐volume co‐incubated with the combination of doxycycline (0–128 µg mL^−1^) with metformin (0–10 mg mL^−1^) at 37 °C for 1 h. Phosphate buffer saline (PBS, 0.01 mol L^−1^, pH = 7.4) in the presence or absence of 0.2% Triton X‐100 was used as a positive control and negative control, respectively. The absorption of released hemoglobin was measured at 576 nm by Infinite M200 Microplate reader (Tecan). Hemolysis rate was determined based on the following formula

(2) $$\begin{array}{ccc} & & \mathrm{Hemolysis}\;\left(\%\right)=\left[\left(OD_{576\;\mathrm{sample}}-OD_{576\;\mathrm{blank}}\right)/\right.\\ & & \left.\left(OD_{576\;0.2\%\;\mathrm{Triton}\;X-100}-OD_{576\;\mathrm{blank}}\right)\right]\times 100\% \end{array}$$

Cytotoxicity on Chinese hamster ovary (CHO) cells was performed by water‐soluble tetrazolium salt‐1 (WST‐1, Roche) assay with the absorbance at 450 nm.^[^ ^66^ ^]^ Doxycycline (0–128 µg mL^−1^) with metformin (0–10 mg mL^−1^) and 1 × 10^4^ cells were simutaneously added in 96‐well plates, and cultured in DMEM supplemented with 10% heat inactivated FBS at 37 °C for 24 h, followed by WST‐1 tests.

##### Time‐Dependent Killing Curve

Overnight culture of *E. coli* B2 was diluted 1:10000 into fresh MHB media and incubated for 4 h (early‐exponential) or 8 h (late‐exponential) at 37 °C under continuous shaking (200 rpm). Then, the culture was challenged by either PBS, doxycycline (2, 32, or 128 µg mL^−1^) or metformin (5 mg mL^−1^) alone or in combination treatment. At the time points 0, 4, 8, 12, and 24 h, 100 µL aliquots were removed, centrifuged, resuspended in PBS, and serially diluted. The dilutions were spotted on MHA agar, and colony counts were determined after overnight incubation at 37 °C. For bactericidal activity analysis, 3 mL of culture at 600 nm (OD_600_) of 1.0 in glass tube was treated with fourfold MIC of doxycycline (128 µg mL^−1^), 5 mg mL^−1^ metformin or their combination for 24 h. All experiments were performed with three biological replicates.

##### Resistance Development Studies

*E. coli* ATCC 25922 at exponential phase were diluted 1:1000 into fresh MHB media supplement with 0.25 × MIC of doxycycline or doxycycline plus 0.25 × MIC of metformin (2.5 mg mL^−1^). After cultured at 37 °C for 24 h, the MIC of culture was determined by twofold serial dilutions in 96‐well microtiter plates. Meanwhile, this culture was diluted into adjusted 0.25 × MIC of drugs for next passages. The process was repeated for 30 d, and the fold increase in MIC of doxycycline relative to initial MIC was calculated.^[^ ^67^ ^]^ Experiments were performed with biological replicates.

##### Fluorescence Assay

In fluorescence assay, *E. coli* B2 and *S. aureus* ATCC 29213 were chosen as the indicator strains. Bacterial pretreatments in all measurements were performed with similar protocols as follows. Briefly, bacteria were grown overnight at 37 °C with shaking at 200 rpm. Then the cultures were washed and suspended with 5 mmol L^−1^ HEPES (pH 7.0, plus 5 mmol L^−1^ glucose). The OD_600_ of bacteria suspension was standardized to 0.5 in same buffer and fluorescent dye was added. After incubation at 37 °C for 30 min, 190 µL of probe‐labeled bacterial cells were added to a 96‐well plate and 10 µL of metformin (final concentrations from 0 to 5 mg mL^−1^) was added. After incubation for 1 h, fluorescence intensity were measured on an Infinite M200 Microplate reader.

##### Outer Membrane Permeability Assay

Fluorescent probe 1‐*N*‐phenylnaphthylamine (NPN) (10 × 10^−6^ m) was used to evaluate the outer membrane integrity of *E. coli* B2 treated by metformin. Fluorescence intensity were measured with the excitation wavelength at 350 nm and emission wavelength at 420 nm.

##### Cell Membrane Integrity Assay

The fluorescent intensity of 10 × 10^−9^ m propidium iodide (PI) in the presence of increasing metformin was measured with the excitation wavelength at 535 nm and emission wavelength at 615 nm.

##### Membrane Depolarization Assay

Bacterial cells were washed and resuspended to obtain an OD_600_ of 0.5 with 5 × 10^−3^ m HEPES (pH 7.0, plus 5 × 10^−3^ m glucose). At the time point of ten min, a final concentration of 3, 3‐dipropylthiadicarbocyanine iodide DiSC_3_(5) (Aladdin, Shanghai, China) (0.5 × 10^−6^ m) was added. After 30 min, final concentration of metformin (5 mg mL^−1^) or doxycycline (8 µg mL^−1^) was injected. Dissipated membrane potential of *E. coli* B2 in the presence two drugs was measured with excitation wavelength at 622 nm and emission wavelength at 670 nm with an interval of 5 min for 40 min. For *S. aureus* ATCC 29213, probe‐labeled cells were incubated with varying concentrations of metformin (0–5 mg mL^−1^) for 60 min, and the fluorescent intensity were determined.

##### Total ROS Measurement

The levels of ROS in *E. coli* B2 treated by metformin was measured with 10 × 10^−6^ m 2′,7′‐dichlorodihydrofluorescein diacetate (DCFH‐DA), following the manufacturer's instruction (Beyotime, Shanghai, China). After incubation for 1 h, the fluorescence intensity was immediately measured with the excitation wavelength at 488 nm and emission wavelength at 525 nm. Rosup was used as a positive control of ROS production.

##### Proton Motive Force Assay

The proton motive force of *E. coli* B2 or *S. aureus* ATCC 29213 treated by metformin was measured with pH‐sensitive fluorescence probe BCECF‐AM (20 × 10^−6^ m). After the fluorescence stabilized, glucose (25 × 10^−6^ m) or varying metformin were added. For all BCECF experiments, the excitation and emission wavelengths on the fluorescence spectrometer were set to 500 and 522 nm, respectively.

##### Efflux Pump Assay

The effect of metformin on the inhibition of efflux pump, EtBr efflux assay was performed based on previous study.^[^ ^68^ ^]^ Cells were co‐incubated with 5 × 10^−6^ m EtBr and sub‐MIC of metformin (5 mg mL^−1^), or known efflux pump inhibitor CCCP (100 × 10^−6^ m) at 37 °C to an OD_600_ of 0.5. After centrifuged at 5000 g at 4 °C g for 10 min, the pellets were collected and resuspended in fresh MHB. Subsequently, EtBr efflux from the cells was monitored with the excitation wavelength at 530 nm and emission wavelength at 600 nm during 60 min.

##### ATP Determination

Intracellular ATP levels of *E. coli* B2 were determined using an Enhanced ATP Assay Kit (Beyotime, Shanghai, China). *E. coli* B2 grown overnight at 37 °C with shaking at 200 rpm were washed and resuspended to obtain an OD_600_ of 0.5 with 0.01 mol L^−1^ PBS (pH 7.4). After treating by various concentrations (0–5 mg mL^−1^) of metformin for 1 h, bacterial cultures were centrifuged at 12 000 g at 4 °C g for 5 min, and the supernatant was removed. Bacterial precipitates were lysed by lysozyme, centrifuged and the supernatant was prepared for intracellular ATP levels measurement. Detecting solution was added to a 96‐well plate and incubated at room temperature for 5 min. Subsequently, the supernatants were added to the well, mixed quickly, and the luminescence was measured by Infinite M200 Microplate reader. Total ATP levels in samples were calculated based on the standard curve of luminescence signals versus concentrations of ATP standard solution.

##### RT‐PCR Analysis

*E. coli* B2 were grown overnight in LB broth and diluted 1/100 into 1 mL fresh LB supplemented with metformin (0 to 5 mg mL^−1^). After bacterial cells were grown to mid‐log phase (OD_600_ = 0.5) at 37 °C, total RNA was extracted using the EASYspin Plus kit (Aidlab, Beijing, China) and quantified by the ratio of absorbance (260 nm/280 nm) using a Nanodrop spectrophotometer (Thermo Scientific, MA, USA). Before cDNA synthesis, RNA from all bacterial cells was adjusted to an identical concentration. Reverse transcription of 1 µg extracted RNA was performed using the PrimeScript RT reagent Kit with gDNA Eraser (Takara, Beijing, China) following the manufacturer's protocol, and 10 ng cDNA (corresponding to 4.63 × 10^8^ copies, calculated through the website: http://cels.uri.edu/gsc/cndna.html) were used as a template for subsequent RT‐PCR tests. Plasmid copy number was calculated by following formula: number of copies = (DNA amount × 6.022 × 10^23^) / (plasmid length × 1 × 10^9^ × 650).^[^ ^69^, ^70^ ^]^

The mRNA levels of *tet*(A) (located on an approximately 20 kb plasmid) relative to the control gene (*rsmC*) in *E. coli* were performed with TB Green qPCR Kit (TaKaRa), according to the optimized primers for *tet*(A) (*for*: GTGAAACCCAACAGACCCCT; *rev*: TGACGTCGTTCGAGTGAACC) and *rsmC* (*for*: GAAATTCTGGGGCGAATACA; *rev*: CTTTCACCTCGGAAAAGACG).^[^ ^47^ ^]^ Thermal cycling was performed by two‐step PCR amplification standard procedure with 95 °C for 30 s and 40 cycles of 95 °C for 5 s, 60 °C for 34 s. RT‐PCR test was performed using 7500 Fast Real‐Time PCR System (Applied Biosystem, CA, USA). The fold changes of gene expression were determined using the 2^−ΔΔCt^ method.

##### Antibiotics Accumulation Analysis

The accumulation of antibiotics in *E. coli* B2 or *S. aureus* ATCC 29213 was determined by LC‐MS/MS analysis according to previous report.^[^ ^71^ ^]^ Briefly, 1.0 mL of an overnight culture of *E. coli* or *S. aureus* was diluted into 100 mL of fresh Luria Bertani (LB) broth and grown at 37 °C with shaking to an optical density (OD_600_) of 0.5. Then bacteria cells were collected and diluted to 10^12^ CFUs mL^−1^ by fresh PBS and aliquoted into 1.5 mL tubes. Doxycycline (32 µg mL^−1^) or kanamycin (1 µg mL^−1^) together with varying metformin (final concentration, 0.5 to 5 mg mL^−1^) were added, then samples were incubated at 37 °C with shaking for 15 min for doxycycline, 10, 20, or 30 min for kanamycin, respectively.

After incubation, bacteria were pelleted by centrifuging at 13 000 g for 2 min. To lyse the samples, each pellet was dissolved in 400 µL of water, then subjected to three freeze‐thaw cycles in liquid nitrogen followed by treatment in water bath at 65 °C. The lysates were pelleted at 13 000 g for 2 min and the supernatants were collected. All debris were re‐suspended in 200 µL of methanol and pelleted as before. The supernatants were combined with the previously collected supernatants. Residual debris was removed by centrifuging at 13 000 g for 10 min. Supernatants were analyzed by on an Agilent 1260 Infinity HPLC system coupled to an AB SCIEX QTRAP 6500 mass spectrometer (ABSciex, CA, USA). The liquid chromatography separation was performed on a C18 column (2.1 × 100 mm, 3 µm) with mobile phase A (0.1% formic acid in water) and mobile phase B (0.1% formic acid in acetonitrile). The flow rate was 0.3 mL min^−1^. The linear gradient was as follows: 0.1–1.0 min, 98% A; 1.0–5.0 min, 98–10% A; 5.0–6.0 min, 10–0% A. 6.0–7.0 min, 0–98%. 7.0–8.0 min, 98% A. The injection volume was 2 µL. The quantification determination of antibiotics uptake was performed by multiple reaction monitoring (MRM) with positive electrospray ionization using the *m/z* 445.3 → 428.3 transition for doxycycline and the *m/z* 485.3 → 163.1 transition for kanamycin.

##### Transcriptomic Analysis

Doxycycline‐resistant *E. coli* B2 were grown in MHB to the exponential phase. Then, cells were incubated with doxycycline (16 µg mL^−1^) alone or in combination of metformin (5 mg mL^−1^) for 4 h. After incubation, cells were harvested and the total RNA of samples was extracted using the EASYspin Plus kit (Aidlab, Beijing, China) and quantified by the ratio of absorbance (260 nm/280 nm) using a Nanodrop spectrophotometer (Thermo Scientific), and sequenced using Hiseq2000 Truseq SBS Kit v3‐HS (200 cycles) (Illumina) with the read length as 2  ×  100 (PE100). Raw sequencing reads were subjected to filtration by quality control, and then mapped against the genome of *E. coli* B2. Further analyses were performed on the free online platform of Majorbio Cloud Platform (Majorbio, Shanghai, China). Differentially expressed genes were identified by gene expression‐level analysis using the FPKM (Fragments Per Kilobase of transcript per Million mapped reads) method with *p*‐values ≤ 0.05 and fold change (FC) values ≥ 2 (log2 FC ≥ 1 or log2 FC ≤ −1). The Cuffdiff program (http://cufflinks.cbcb.umd.edu/) was used to analyze differences between these two treatments.

##### Intracellular Bacteria Determination

Vero cells were infected with *E. coli* B2 cells at an MOI of 100, and co‐culture with doxycycline (0–256 µg mL^−1^) or in combination with metformin (5 mg mL^−1^) for 6 h at 37 °C in 5% CO_2_. Extracellular bacteria were removed by colistin (50 µg mL^−1^) incubated for 15 min and washed with PBS twice. Subsequently, cells lysed by DMEM supplemented with 0.1% BSA and 0.1% Triton X‐100, and serial dilutions of the lysates were plated on MHA for CFU counting.

##### Cytokines Measurement

The production of cytokines of TNF‐*α*, IL‐1*β*, IL‐8, and IFN‐*γ* in RAW264.7 culture supernatants were measured by commercially available enzyme‐linked immunosorbent assay (ELISA) kits (Beyotime, Shanghai, China) according to the manufacturer's instructions.

##### Galleria Mellonella Infection Model

The synergy between doxycycline and metformin was evaluated in the *Galleria mellonella* larvae infection model. The larvae of *Galleria mellonellas* (Huiyude Biotech Company, Tianjin, China) were randomly divided into four groups (*n* = 10 per group) and infected with 10 µL of *E. coli* B2 suspension (1.0 × 10^6^ CFUs) at the right posterior gastropoda. After 2 h postinfection, *Galleria mellonella* were treated with either PBS as vehicle, doxycycline (50 mg kg^−1^) or metformin (50 mg kg^−1^) alone or in combination (50 + 50 mg kg^−1^) at left posterior gastropoda. Survival rates of G*alleria mellonella* larvae were recorded during 120 h.

##### Animal Usage Declaration

6–8 week old female BALB/c mice were obtained from Center of Comparative Medicine in Yangzhou University. Mice were adapted to standardized environmental conditions (temperature = 23 ± 2 °C; humidity = 55 ± 10%) for one week prior to infection. Mice were maintained in strict accordance with the regulations for the Administration of Affairs Concerning Experimental Animals approved by the State Council of People's Republic of China (11‐14‐1988). The animal study protocols were performed in accordance with the relevant guidelines and regulations (ID: SCYK2017‐0007). The laboratory animal usage license number is SYXK‐2017‐0044, certified by Jiangsu Science and Technology Department.

##### Mouse Peritonitis Infection Model

Female BALB/C mice (*n* = 8 per group) were intraperitoneally infected with a lethal dose of 1.0 × 10^8^ CFUs *E. coli* B2 suspension. After 2 h postinfection, mice were treated with a single dose of doxycycline (50 mg kg^−1^), metformin (50 mg kg^−1^) alone or a combination of doxycycline plus metformin (50 + 50 mg kg^−1^) via intraperitoneal injection. Survival rates of treated mice were recorded during 7 d.

##### Neutropenic Mouse Thigh Infection Model

Female BALB/C mice (*n* = 8 per group) were firstly rendered neutropenic by cyclophosphamide (two consecutive doses of 150 and 100 mg kg^−1^ delivered on 4 and 1 d before infection).^[^ ^72^ ^]^ Then, 100** **µL of *E. coli* B2 bacterial suspension (1.0 × 10^6^ CFUs per mouse) or MRSA T144 suspension (1.5 × 10^5^ CFUs per mouse) were injected into the right thighs of each mouse. At 2 h post infection, doxycycline (50 mg kg^−1^) or metformin (50 mg kg^−1^) alone or in combination (50 + 10 or 50 + 50 mg kg^−1^) were given by intraperitoneal injections. At 48 h postinfection, mice were euthanized by cervical dislocation. The right thighs were aseptically removed, homogenized, serially diluted, and plated on MHA to count bacterial numbers after incubated at 37 °C for 24 h.

##### Statistical Analyses

Statistical analysis was performed using GraphPad Prism 6 and SPSS software. All data were presented as means ± SD. Unless otherwise noted, unpaired *t*‐test between two groups or one‐way ANOVA among multiple groups were used to calculate *p*‐values (*p < 0.05, **p < 0.01, ***p < 0.001).

## Conflict of Interest

The authors declare no conflict of interest.

## Author Contributions

Z.W., K.Z., and Y.L. designed and conceived the project. Y.L., Y.J., and K.Y. performed experiments. Y.L., R.L., and X.X. analyzed the data. Y.L., K.Z., and Z.W. wrote the manuscript. All authors read and approved the manuscript.

## Supporting information

Supporting information
