# Supplementary material for: Metformin Restores Tetracyclines Susceptibility against Multidrug Resistant Bacteria
Source: Adv Sci (Weinh). 2020 May 12;7(12):1902227. doi: 10.1002/advs.201902227 (PMC7312304; doi:10.1002/advs.201902227)
Supplement: Supplementary file 1 — Supporting information [file ADVS-7-1902227-s001.pdf]

# Supporting information

## Metformin restores tetracyclines susceptibility against multi-drug resistant bacteria

Yuan Liu[1,2], Yuqian Jia[1], Kangni Yang[1], Ruichao Li[1,2], Xia Xiao[1,2], Kui Zhu[3]*, Zhiqiang Wang[1,2]*

[1]Institute of Comparative Medicine, College of Veterinary Medicine, Yangzhou University, Yangzhou, Jiangsu, China

[2]Jiangsu Co-innovation Center for Prevention and Control of Important Animal Infectious Diseases and Zoonoses, Yangzhou, Jiangsu, China

[3]Beijing Advanced Innovation Center for Food Nutrition and Human Health, College of Veterinary Medicine, China Agricultural University, Beijing, China

**Correspondence author:** zqwang@yzu.edu.cn (Z. W.) and zhuk@cau.edu.cn (K. Z.)

## Figures

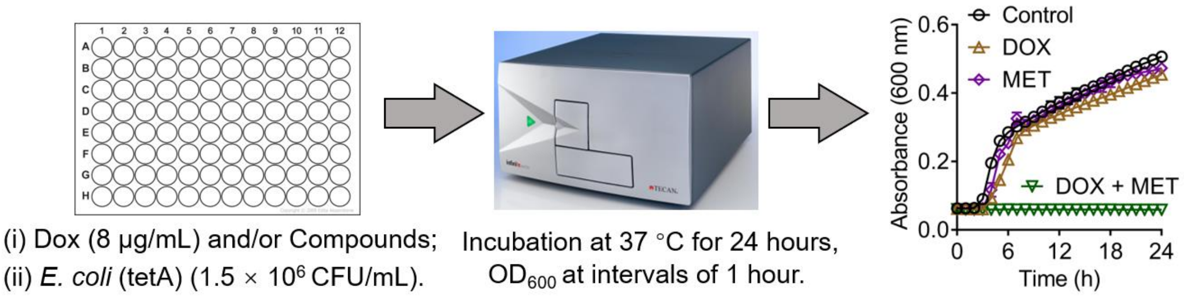


**Figure S1 Scheme of screening of novel tetracyclines adjuvants from FDA-approved compounds.** 158 FDA-approved compounds from Prestwick Chemical Library were screened against *E. coli* B2 (*tet*(A)) in combination with 8 μg/mL doxycycline (one quarter of the minimum inhibitory concentration). Briefly, doxycycline and/or compounds were diluted in Mueller-Hinton Broth (MHB) and mixed with an equal volume of bacterial suspensions containing approximately 1.5 × $10^6$ colony-forming units (CFUs)/mL in a clear UV-sterilized 96-well microliter plate. Then, the real-time growth curves of *E. coli* B2 in the absence or presence of drugs were monitored during 24 hours. Compounds that significant inhibits bacterial growth (inhibition rate ≥ 50%) in combination treatment, whereas no direct antibacterial activity in monotreatment were defined as the potential tetracyclines adjuvants candidates.

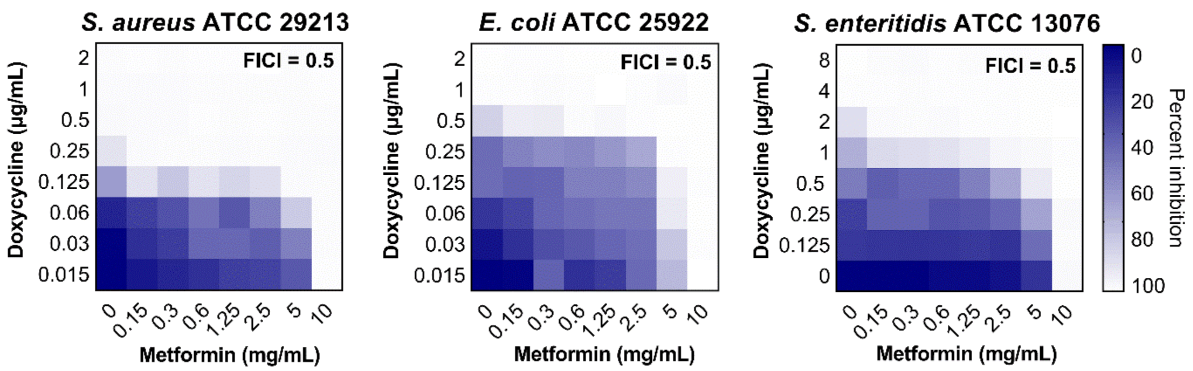


**Figure S2 Weak synergistic activity between metformin and doxycycline against sensitive bacteria.**

Dark blue regions represent higher cell density and lower inhibition rate. Data represent the mean OD (600 nm) of two biological replicates.

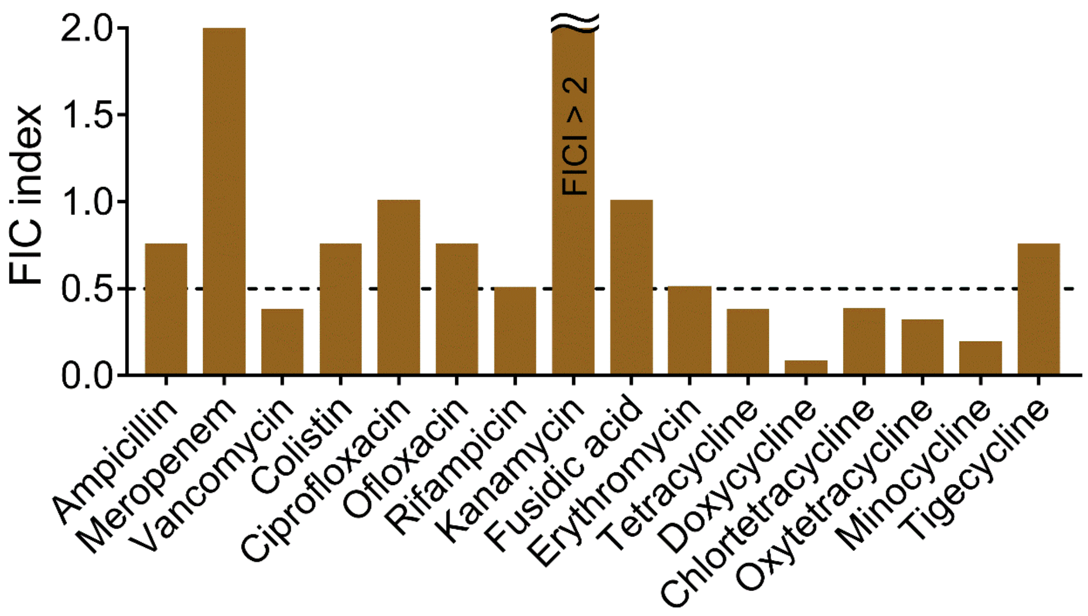


**Figure S3 FIC indices of metformin and different classes of antibiotics against *E. coli* B2.**

FIC indices were calculated based on chequerboard microdilution assays at 0.25 or 0.125 × MIC (only for kanamycin) of metformin. Synergy is defined as an FIC index of ≤ 0.5.

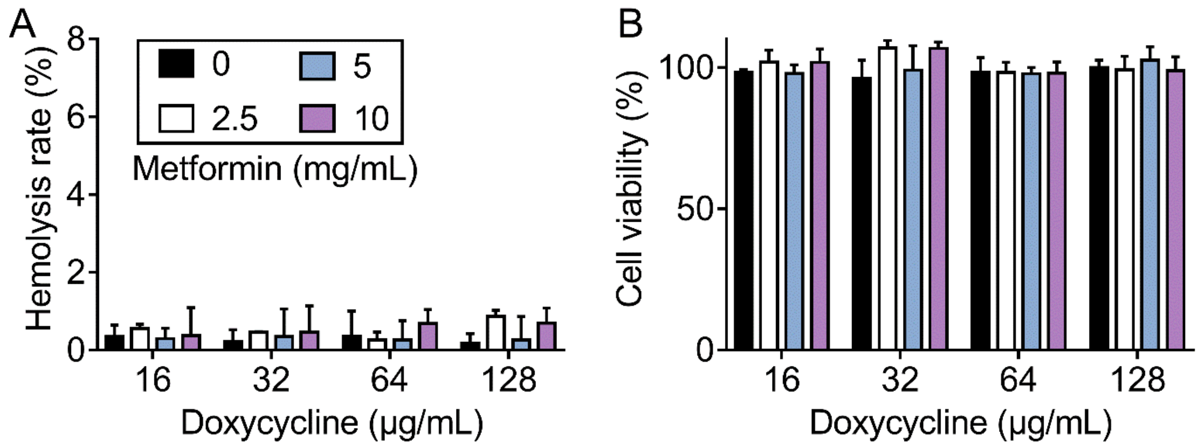


**Figure S4 Effect of metformin on the safety of doxycycline.**

**(A)** Hemolytic activity of doxycycline to the RBCs in the absence or presence of metformin.

**(B)** Addition of metformin exerts negligible effect on the cytotoxicity of doxycycline in Chinese hamster ovary (CHO) cells.

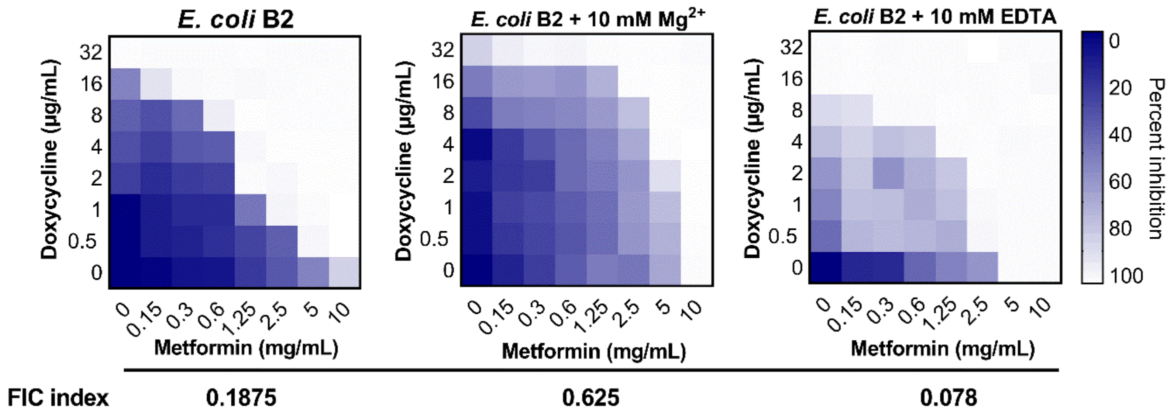


**Figure S5 Effect of exogenous $Mg^{2+}$ or EDTA on the synergistic activity between doxycycline and metformin.**

The addition of $Mg^{2+}$ (10 mM) abolished the potentiation activity of metformin to doxycycline, whereas EDTA enhanced their synergy effect. Dark blue regions represent higher cell density and lower inhibition rate. Data represent the mean OD (600 nm) of two biological replicates.

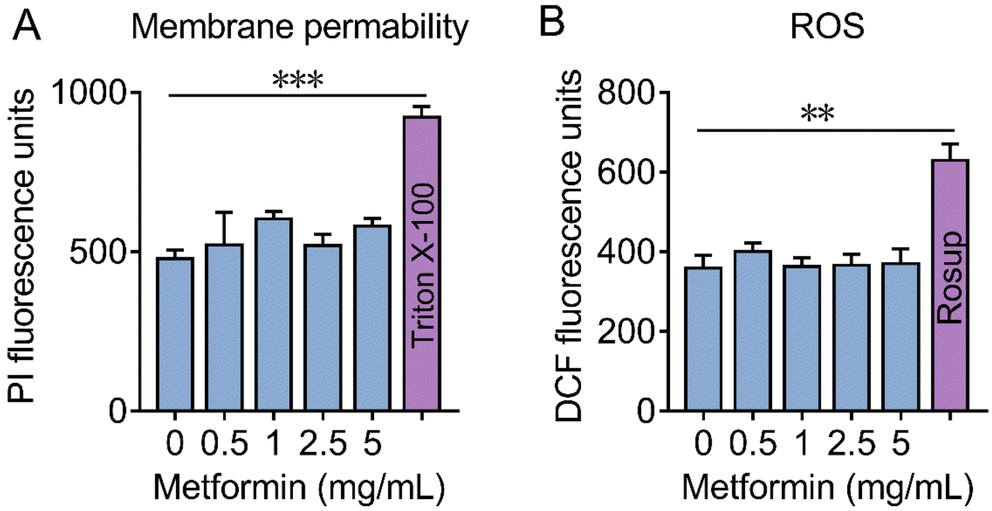


**Figure S6 Metformin has no effect on the membrane permeability and ROS production of *E. coli* B2.**

**(A)** No significant effect on the whole membrane permability of *E. coli* B2 under the stimulation of varying metformin for 1 h, probed by propidium iodide (10 nM).

**(B)** None of ROS production by metformin were determined. probed with 2’,7’-dichlorodihydrofluorescein diacetate (DCFH-DA). Rosup was used as the positive control of ROS production.

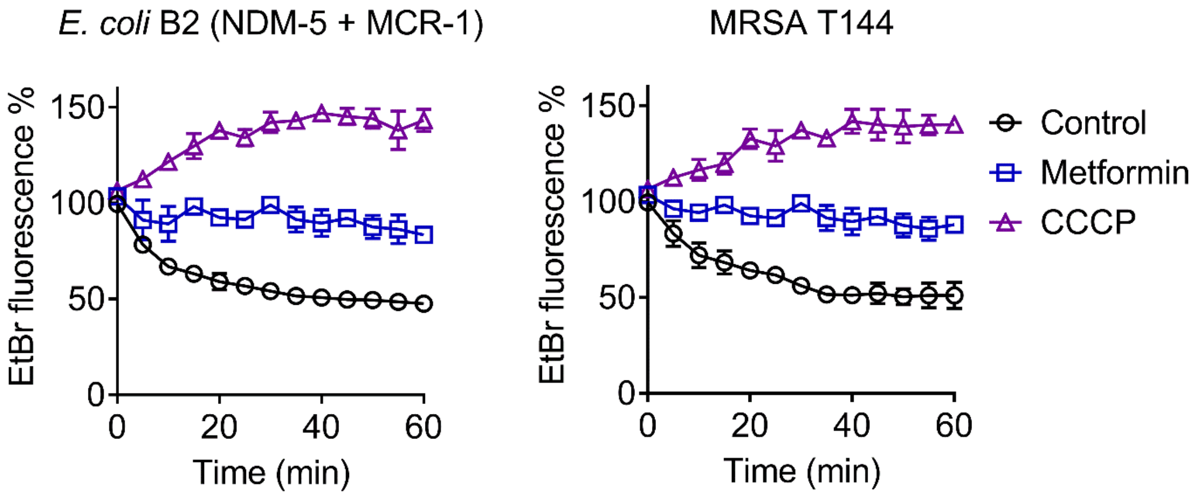


**Figure S7 Inhibition of efflux pump by metformin or CCCP in *E. coli* B2 and MRSA T144.**

Cells were co-incubated with 5 µM EtBr and sub-MIC of metformin (5 mg/mL), or known efflux pump inhibitor CCCP (100 µM) at 37 °C to an $OD_{600}$ of 0.5. Then, EtBr efflux from the cells was monitored with the excitation wavelength at 530 nm and emission wavelength at 600 nm during 60 minutes.

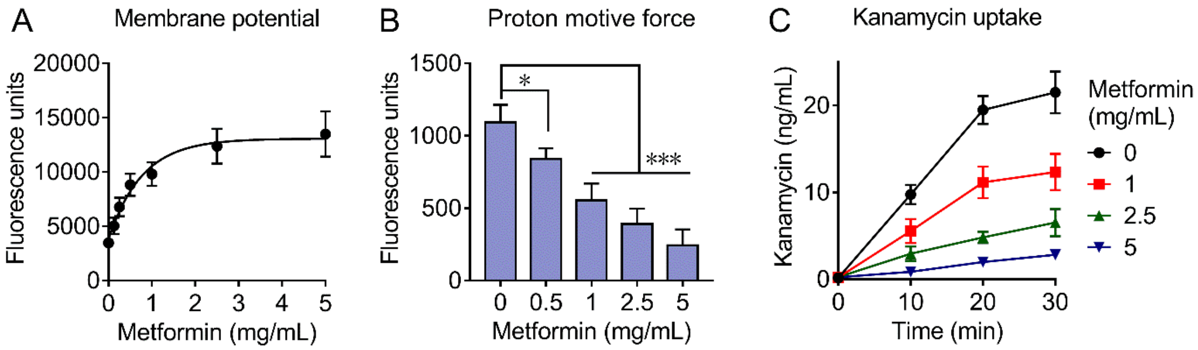


**Figure S8 Metformin collapses proton motive force in *S. aureus* ATCC 29213.**

**(A)** Metformin dissipates membrane potential of *S. aureus*. Membrane potential was determined by measuring the fluorescence of $DiSC_3(5)$ after 60 min exposure to increasing concentrations of metformin.

**(B and C)** Metformin decreased proton motive force (B) and thereby reduced the kanamycin uptake (C) in *S. aureus* in a dose-dependent manner. Proton motive force of *S. aureus* after exposing varying concentration of metformin were determined by monitoring BCECF fluorescence intensity. Kanamycin uptake in *S. aureus* were determined by LC-MS/MS. All data was presented as means ± SD and one-way ANOVA were used to calculate *P*-values (**P* < 0.05, ****P* < 0.001).

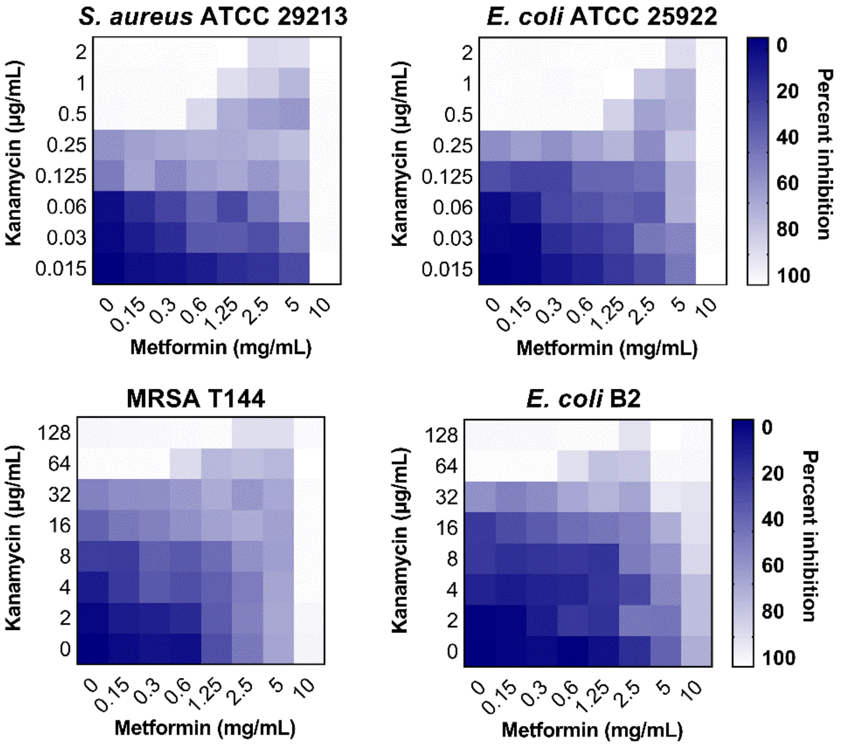


**Figure S9 Combination of metformin and kanamycin leads to antagonistic interactions against sensitive bacteria (*S. aureus* ATCC 29213 and *E. coli* ATCC 25922) and resistant bacteria (MRSA T144 and *E. coli* B2).**

Data were obtained by chequerboard broth microdilution assays and shown as a heat plot. Dark blue regions represent higher cell density and lower inhibition rate of combination treatment.

# Tables

**Table S1 Bacterial strains used in this study.**

| Organism and genotype | Source/Reference |
|---|---|
| **Gram-positive bacteria** | |
| *Enterococcus faecalis* A4 (*tet*(A), *van*A, $Van^R$) | [1] |
| *Staphylococcus aureus* ATCC 29213 | ATCC |
| *S. aureus* 215 ($LZD^R$, *cfr*, *tet*(A)) | [2] |
| MRSA T144 (*mecA*, *tet*(A)) | [2] |
| **Gram-negative bacteria** | |
| *Escherichia coli* ATCC 25922 | ATCC |
| *E. coli* B2 (*tet*(A), $bla_{NDM-5}$, $bla_{TEM-1B}$, $bla_{OXA-10}$, $bla_{CTX-M-14}$, *mcr-1*, *aadA*, *aph*(4), *oqxAB*, *mdfA*, *fosA3*) | [3] |
| *Salmonella enteritidis* ATCC 13076 | ATCC |
| *S. enteritidis* H8 (*tet*(A), $bla_{NDM-1}$) | [3] |

ATCC, American Type Culture Collection; $Van^R$, vancomycin resistance; $LZD^R$, linezolid resistance.

## References


[1] Y. Liu, Y. Jia, K. Yang, R. Li, X. Xiao, Z. Wang, *ACS Infectious Diseases* **2019**, DOI: 10.1021/acsinfecdis.9b00164.

[2] Y. Liu, S. Ding, R. Dietrich, E. Märtlbauer, K. Zhu, *Angew. Chem. Int. Ed.* **2017**, *56*, 1486.

[3] Y. Liu, K. Yang, Y. Jia, Z. Wang, *ACS Infectious Diseases* **2019**, *5*, 2061.

**Table S2 Interaction between 158 FDA-approved compounds with doxycycline against *E. coli* B2.**

| Compounds | Inhibition rate (%)[#] | Compounds | Inhibition rate (%)[#] | Compounds | Inhibition rate (%)[#] |
|---|---|---|---|---|---|
| Acetylcholine | 33.58 ± 5.48 | Disulfiram | 69.81 ± 7.44 | Oxiracetam | 12.56 ± 0.13 |
| Adenine | 38.21 ± 10.96 | Ebselen | 26.31 ± 12.42 | Oxolinic acid | 24.22 ± 7.95 |
| Amikacin | 37.69 ± 7.51 | EGCG | 78.36 ± 1.03 | Oxyfedrine | 50.16 ± 6.55 |
| Amoxicillin | -9.35 ± 0.72 | Enoxacin | 6.72 ± 11.88 | Paraquat | 35.68 ± 7.2 |
| Anethole | 38.08 ± 1.56 | Enrofloxacin | 20.16 ± 14.09 | Paromomycin | 16.09 ± 8.23 |
| Anisindione | 21.08 ± 0.53 | Erythromycin | 37.79 ± 13.8 | Pefloxacine | 1.82 ± 10.76 |
| Apramycin | 6.87 ± 7.97 | Estradiol | 27.92 ± 5.86 | Penicillin G | -4.9 ± 14.38 |
| Artemisinin | 10.54 ± 7.72 | Estriol | 29.3 ± 3.97 | Phenformin | 38.85 ± 12.13 |
| Artesunate | 18.49 ± 13.88 | Estrone | 11.48 ± 1.63 | Phenindione | 6.15 ± 12.84 |
| Ascorbic acid | 15.91 ± 11.54 | Estropipate | 31.41 ± 4.75 | Phleomycin | 10.39 ± 3.69 |
| Aspirin | 7.33 ± 0.91 | Ethacrynic acid | 25.05 ± 1.21 | Pipemidic acid | 31.89 ± 6.3 |
| Auranofin | 37.81 ± 9.17 | Fleroxacin | 38.4 ± 12.83 | Piperacillin | -13.59 ± 9.89 |
| Azacytidine-5 | 8.91 ± 3.81 | Florfenicol | 36.94 ± 7.46 | Piperazine | 18.07 ± 13.92 |
| Azidothymidine | 13.89 ± 2.75 | Floxuridine | 3.92 ± 11.78 | Piretanide | 6.89 ± 4.04 |
| Azithromycin | 6.72 ± 8.47 | Fludarabine | 28.96 ± 6.83 | Pivmecillinam | 8.03 ± 10.39 |
| Aztreonam | 3.52 ± 6.33 | Flumequine | 38.08 ± 11.04 | Procaine | 32.24 ± 9.77 |
| Bacitracin | 4.61 ± 14.54 | Fluspirilen | 22.69 ± 12.89 | Pseudomonic acid | -8.95 ± 6.6 |
| Baicalein | 38.81 ± 2.02 | Fosfomycin | -3.52 ± 11.8 | Pterostilbene | 5.59 ± 14.28 |
| Benserazide | 80.75 ± 8.95 | Furazolidone | 3.67 ± 3.12 | Pyocyanin | -15.35 ± 2.46 |
| Benzalkonium | 24.88 ± 12.24 | Fusidic acid | 38.45 ± 4.81 | Pyrimethamine | 19.8 ± 14.68 |
| Benzydamine | 73.86 ± 2.18 | Gatifloxacin | 30.98 ± 13.11 | Reserpine | 41.72 ± 5.4 |
| Berberine | 9.36 ± 4.32 | Gentamicin | 6.58 ± 2.04 | Rifabutin | 8.22 ± 2.49 |
| Beta-thujaplicin | 68.97 ± 14.86 | Glutamate | 6.03 ± 4.61 | Rifampicin | 9.14 ± 14.1 |
| Caffeine | 9.44 ± 13.08 | Glycine | 25.21 ± 12.1 | Rifapentine | 4.48 ± 0.82 |
| Carbenicillin | -1.11 ± 2.78 | Grepafloxacin | 24.9 ± 8.99 | Rifaximin | 12.48 ± 0.54 |
| Cefepime | -6.67 ± 8.24 | Guanine | 36.77 ± 10.33 | Ronidazole | 1.79 ± 3.26 |
| Cefotaxime | -0.98 ± 7.22 | Hexachloro-phene | 23.42 ± 4.12 | Roxithromycin | 8.63 ± 4.83 |
| Cefquinome | -4.69 ± 3.37 | Indole | 33.12 ± 4.3 | Rufloxacin | 20.15 ± 2.27 |
| Ceftazidime | -6.77 ± 0.58 | Kanamycin | 7.99 ± 11.97 | Sarafloxacin | 26.85 ± 5.38 |
| Ceftibuten | 4.79 ± 4.91 | Levofloxacin | 3.72 ± 9.11 | Spectinomycin | 39.62 ± 5.13 |
| Ceftiofur | -5.68 ± 2.67 | Lomefloxacin | 37.6 ± 2.65 | Streptozotocin | 4.13 ± 3.66 |
| Cerulenin | -13.66 ± 8.43 | Loperamide | 83.65 ± 5.81 | Sulfamonome-thoxine | 8.19 ± 12.58 |
| Chloramph-enicol | 27.09 ± 11.21 | Lovastatin | 23.67 ± 4.34 | Tazobactam | 36.87 ± 7.52 |
| Chlorhexidine | 55.17 ± 5.34 | Mecillinam | -16.79 ± 14.32 | Tegaserod | 66.17 ± 13.4 |
| Chlorotropone | 5.53 ± 7.97 | Meclocycline sulfosalicylate | 8.2 ± 11.99 | Theophylline | 14.32 ± 14.29 |

| | | | | | |
|---|---|---|---|---|---|
| Chloroxine | 65.98 ± 5.75 | Melatonin | 20.99 ± 10.55 | Thioguanosine | 3.74 ± 4.49 |
| Chlortetracy-cline | 25.4 ± 7.62 | Merbromin | 0.67 ± 10.72 | Thiostrepton | 37.06 ± 2.45 |
| Ciclopirox ethanolamine | 9.62 ± 10.99 | Meropenem | 25.57 ± 10.37 | Thonzonium bromide | 37.16 ± 4.31 |
| Ciprofloxacin | 15.91 ± 5.9 | Metformin | 93.41 ± 2.51 | Thymine | 15.2 ± 12.85 |
| Clarithromycin | 62.17 ± 11.08 | Methicillin | -1.06 ± 1.51 | Ticarcillin sodium | 29.4 ± 0.52 |
| Clavulanate | 27.68 ± 6.98 | Methoxy-tropone | 3.87 ± 9.05 | Tobramycin | 62.15 ± 1.1 |
| Clinafloxacin | 33.16 ± 9.91 | Mitomycin C | 42.21 ± 7.15 | Tosufloxacin | 5.74 ± 14.69 |
| Clioquinol | 0.44 ± 8.6 | Moxifloxacin | 17.39 ± 7.96 | Triclosan | 38.61 ± 7.42 |
| Clofazimine | 16.92 ± 2.45 | Nisin | 30.81 ± 15 | Trimethoprim | -18.08 ± 6.53 |
| Colistin | 21.7 ± 14.33 | Nitrofurantoin | 21.99 ± 8.45 | Tropolone | 80.78 ± 11.58 |
| Curcumin | 12.68 ± 2.2 | Novobiocin | 28.74 ± 3.19 | Tropone | 28.22 ± 12.8 |
| Cycloserine D | 15.77 ± 0.57 | Ofloxacin | 37.42 ± 13.74 | Tryptophan | 6.43 ± 14.61 |
| Cytosine | 33.3 ± 13.67 | Oxacillin | -0.93 ± 14.87 | Uracil | 6.62 ± 3.73 |
| Dapsone | 26.99 ± 1.44 | Oxantel pamoate | 24.88 ± 10.16 | Vancomycin | 21.64 ± 5.27 |
| Demeclocycline | 33.01 ± 8.57 | Oxaprozin | 33.14 ± 11.49 | Vanillin | -17.18 ± 14.08 |
| Didanosine | 37.75 ± 7.39 | Oxatomide | 30.99 ± 4.25 | Verapamil | 66.22 ± 0.75 |
| Dihydrostrep-tomycin | 7.58 ± 6.31 | Oxcarbazepine | 22.88 ± 4.82 | Oxiracetam | 12.56 ± 0.13 |
| Dirithromycin | 37.66 ± 10.04 | Oxethazaine | 34.76 ± 5.34 | | |

#Data are representative of three independent experiments ± SD. Synergy effect were defined as the inhibition rate of ≥ 50% and marked by green background.

**Table S3 Antimicrobial susceptibility test of strains used in this study (MIC, μg/mL).**

| Organism and genotype | Ampicillin | Vancomycin | Colistin | Doxycycline | Tigecycline |
|---|---|---|---|---|---|
| **Gram-positive bacteria** | | | | | |
| *S. aureus* ATCC 29213 | 0.25 | 0.5 | 16 | 0.25 | 0.125 |
| MRSA T144 | 32 | 2 | 128 | 16 | 2 |
| *S. aureus* 215 | 64 | 1 | 64 | 16 | 1 |
| *E. faecalis* A4 | 32 | >128 | 128 | 32 | 0.125 |
| **Gram-negative bacteria** | | | | | |
| *E. coli* ATCC 25922 | 8 | 128 | 0.5 | 1 | 0.125 |
| *E. coli* B2 | >128 | 128 | 8 | 32 | 2 |
| *S. enteritidis* ATCC 13076 | 8 | 128 | 0.25 | 4 | 0.125 |
| *S. enteritidis* H8 | 8 | 128 | 0.25 | 16 | 0.125 |

ATCC, American Type Culture Collection.

**Table S4 Adjuvant potency of metformin in combination with doxycycline against multi-drug resistant and sensitive bacteria.**

| Pathogens | MIC[a] (μg/mL) | FIC index | MIC[b] (μg/mL) | Potentiation (fold)[c] |
|---|---|---|---|---|
| **Resistant** | | | | |
| MRSA T144 | 16 | 0.091 | 0.5 | 32 |
| *S. aureus* 215 | 16 | 0.310 | 0.5 | 32 |
| VRE A4 | 32 | 0.313 | 1 | 32 |
| *E. coli* B2 | 32 | 0.188 | 0.5 | 64 |
| *S. enteritidis* H8 | 16 | 0.208 | 0.5 | 32 |
| **Sensitive** | | | | |
| *S. aureus* 29213 | 0.5 | 0.5 | 0.125 | 4 |
| *E. coli* 25922 | 1 | 0.5 | 0.25 | 4 |
| *S. enteritidis* 13076 | 4 | 0.5 | 1 | 4 |

[a/b]MICs of doxycycline in the absence or presence of 0.25 × MIC metformin.

[c]Degree of doxycycline potentiation in the presence of 0.25 × MIC metformin.

**Table S5 Synergistic activity of metformin in combination with different classes of antibiotic against *E. coli* B2.**

| Targets | Antibiotics | MIC[a] (μg/mL) | FIC index | MIC[a] (μg/mL) | Potentiation (fold)[c] |
|---|---|---|---|---|---|
| Cell wall | Ampicillin | 128 | 0.75 | 32 | 4 |
| | Meropenem | 32 | 2 | 32 | – |
| | Vancomycin | 128 | 0.375 | 64 | 4 |
| Cell membrane | Colistin | 8 | 0.75 | 2 | 4 |
| DNA synthesis | Ciprofloxacin | 32 | 1 | 16 | 2 |
| | Ofloxacin | 32 | 0.75 | 16 | 2 |
| RNA synthesis | Rifampicin | 128 | 0.5 | 32 | 4 |
| Protein | Kanamycin[d] | 64 | >2 | >128 | – |
| | Fusidic acid | 8 | 1 | 4 | 2 |
| | Erythromycin | 128 | 0.5 | 64 | 4 |
| | Tetracycline | 128 | 0.375 | 16 | 8 |
| | Doxycycline | 32 | 0.078 | 0.5 | 64 |
| | Chlortetracycline | 128 | 0.378 | 32 | 4 |
| | Oxytetracycline | 256 | 0.313 | 64 | 4 |
| | Minocycline | 16 | 0.188 | 1 | 16 |
| | Tigecycline | 2 | 0.75 | 1 | 2 |

[a/b]MICs of antibiotic in the absence or presence of 5 mg/mL metformin (0.25 × MIC).

[c]Degree of antibiotic potentiation in the presence of 5 mg/mL metformin (0.25 × MIC).

[d]For kanamycin, 0.125 × MIC of metformin was used to calculate its FIC index and potentiation fold in *E. coli* B2.

–, none of potentiation activity.
